# *Defective kernel 66* encodes a GTPase essential for kernel development in maize

**DOI:** 10.1093/jxb/erad289

**Published:** 2023-07-25

**Authors:** Yi Ming Wei, Bo Hui Wang, Dong Jie Shao, Ru Yu Yan, Jia Wen Wu, Guang Ming Zheng, Ya Jie Zhao, Xian Sheng Zhang, Xiang Yu Zhao

**Affiliations:** State Key Laboratory of Crop Biology, College of Life Sciences, Shandong Agricultural University, Taian, Shandong 271018, China; College of Life Sciences, Zaozhuang University, Zaozhuang, Shandong 277160, China; State Key Laboratory of Crop Biology, College of Life Sciences, Shandong Agricultural University, Taian, Shandong 271018, China; State Key Laboratory of Crop Biology, College of Life Sciences, Shandong Agricultural University, Taian, Shandong 271018, China; College of Life Sciences, Zaozhuang University, Zaozhuang, Shandong 277160, China; State Key Laboratory of Crop Biology, College of Life Sciences, Shandong Agricultural University, Taian, Shandong 271018, China; State Key Laboratory of Crop Biology, College of Life Sciences, Shandong Agricultural University, Taian, Shandong 271018, China; State Key Laboratory of Crop Biology, College of Life Sciences, Shandong Agricultural University, Taian, Shandong 271018, China; State Key Laboratory of Crop Biology, College of Life Sciences, Shandong Agricultural University, Taian, Shandong 271018, China; State Key Laboratory of Crop Biology, College of Life Sciences, Shandong Agricultural University, Taian, Shandong 271018, China; State Key Laboratory of Crop Biology, College of Life Sciences, Shandong Agricultural University, Taian, Shandong 271018, China; University of Sydney, Australia

**Keywords:** Endosperm, GTPase, kernel development, maize, mitochondria, mitochondrial ribosome

## Abstract

The mitochondrion is a semi-autonomous organelle that provides energy for cell activities through oxidative phosphorylation. In this study, we identified a *defective kernel 66* (*dek66*)-mutant maize with defective kernels. We characterized a candidate gene, *DEK66*, encoding a ribosomal assembly factor located in mitochondria and possessing GTPase activity (which belongs to the ribosome biogenesis GTPase A family). In the *dek66* mutant, impairment of mitochondrial structure and function led to the accumulation of reactive oxygen species and promoted programmed cell death in endosperm cells. Furthermore, the transcript levels of most of the key genes associated with nutrient storage, mitochondrial respiratory chain complex, and mitochondrial ribosomes in the *dek66* mutant were significantly altered. Collectively, the results suggest that DEK66 is essential for the development of maize kernels by affecting mitochondrial function. This study provides a reference for understanding the impact of a mitochondrial ribosomal assembly factor in maize kernel development.

## Teaser

*DEK66*encodes a mitochondrial ribosomal assembly factor and is involved in the regulation of maize kernel development by affecting the morphology and function of mitochondria.

## Introduction

As a sink organ for starch, proteins, lipids, and essential micronutrients such as vitamins and minerals, the maize (*Zea mays* L.) kernel is the most important component for improving yield and quality of maize (Wu and Messing, 2014; Yang *et al.*, 2017; Hu *et al.*, 2021). Maize kernels are mainly composed of three distinct compartments, namely endosperm, embryo, and pericarp, accounting for 83%, 11%, and 6% of the whole dry kernel weight, respectively (Nuss and Tanumihardjo, 2010). The mature endosperm mainly consists of seven types of cells, namely the basal endosperm transfer layer (BETL), aleurone layer (AL), subaleurone, starchy endosperm, basal intermediate zone, conducting zone, and embryo-surrounding region (Zhan *et al.*, 2015; Doll *et al.*, 2020). Differentiation of endosperm cells is very important for grain development and yield formation in maize.

Mitochondria produce cellular energy in the form of ATP through oxidative phosphorylation (OXPHOS), which is the main source of energy for plant development and morphogenesis (Sweetlove *et al.*, 2007). The mitochondrial genome of plants is very large (usually 200–750 kb), but the proteins encoded by it only account for a small part of the total mitochondrial proteins (Gualberto *et al.*, 2014). Approximately 95% of total mitochondrial proteins are encoded in the nucleus, synthesized in the cytoplasm, transported to mitochondria, and assembled with other mitochondrial proteins to form mitochondrial respiratory complexes and other functional proteins. The co-ordination of expression of mitochondrial genes is essential for regulating the OXPHOS capacity in response to both physiological demands and environmental signals (Small *et al.*, 2020). The maintenance of mitochondrial function plays a crucial role in maize kernel development (Wang *et al.*, 2017; Li *et al.*, 2018; Cao *et al.*, 2022). Containing a large number of non-coding sequences, the maize mitochondrial genome only encodes 35 known proteins, three rRNAs, and 21 tRNAs (Clifton *et al.*, 2004) despite its size of approximately 569 kb.

The formation of functional mitochondria requires the proper assembly of ribosomes and coordinated expression of mitochondrial genes. Damaged mitochondrial ribosomes have varied effects on mitochondrial gene translation, and eventually directly affects the assembly of respiratory chain complexes, resulting in impaired mitochondrial structure and function (Janska and Kwasniak, 2014). Previous studies have reported that mitoribosomal proteins are involved in the regulation of maize kernel development and nutrient accumulation. Maize *defective kernel 44* (*DEK44*) encodes a putative mitochondrial 50S ribosomal protein, namely the L9 ribosomal protein. The loss of DEK44 function in maize strongly affects the biogenesis and morphology of mitochondria and results in small kernels with embryo-lethal phenotypes (Qi *et al.*, 2019). Nonzein protein 1 (NZP1), a mitochondrial ribosomal protein, encodes the 50S ribosomal protein L10, which is localized in both mitochondrial ribosomes and protein bodies in maize. Similar to other mutations affecting mitochondrial proteins, *NZP1* impairs mitochondrial function and morphology and affects normal seed development and protein accumulation in maize (Feng *et al.*, 2022).

Compared with bacterial ribosomes, plant mitochondrial ribosomal proteins and rRNA have undergone various changes in structure and function during their evolution, which has increased the complexity of mitochondrial ribosomal assembly (Waltz *et al.*, 2019). Studies have reported that GTPases may play a key role in ribosomal assembly in bacteria and eukaryotes. Ribosome biogenesis GTPase A (RbgA) is a generally conserved protein that is required for the growth of *Bacillus subtilis* (Uicker *et al.*, 2006). Moreover, RbgA can recruit ribosomal proteins L16, L27, and L36 to participate in the assembly of 50S ribosomal subunits at the late stage of ribosome biosynthesis (Matsuo *et al.*, 2006; Uicker *et al.*, 2006; Britton, 2009). However, current studies on the genes related to mitochondrial ribosomal proteins in plants mainly focus on the biological processes regulated by them, such as embryonic development, vegetative growth, and reproductive development (Robles and Quesada, 2017). The molecular and cytological processes in which mitochondrial ribosomal proteins are involved are rarely studied. Therefore, the mechanism of mitochondrial ribosomal assembly and translation of mitochondrial ribosomal proteins in seed development is still unclear.

In this study, we identified the *defective kernel 66* (*dek66*) mutant of maize and characterized the candidate gene, *DEK66*, which is involved in kernel development. Map-based cloning revealed that a point mutation in *dek66* was responsible for the mutant phenotype, which was verified by an allelism test between two types of *dek66* allelic mutants and transgenic complementation of *dek66*. *DEK66* encodes a ribosomal assembly factor located in mitochondria. Functional analysis indicated that the maintenance of mitochondrial structure and function is vital for kernel development in maize.

## Materials and methods

### Plant materials

The *dek66* mutant of maize was obtained by screening an ethyl methane sulfonate (EMS)-mutagenized B73 F_2_ population. To purify the background of the mutants, *dek66* heterozygous plants were continuously crossed to B73 and self-pollinated to generate BC_5_ F_1_ plants before phenotypic analysis. Further, the *dek66* mutant was crossed into an S162 genetic background, and the progeny was self-pollinated to develop an F_2_ segregating population for phenotyping and gene mapping.

### Cytological sectioning

Wild-type (WT) and mutant kernels were sampled at various developmental stages from self-pollinated ears in the *dek66* heterozygous plants. The kernels were cut along the longitudinal axis into three equal sections, and the center section samples were fixed in an FAA fixative solution (5% formalin, 50% ethanol, and 5% acetic acid) overnight at 4 °C. The fixed samples were dehydrated in a graded concentration of ethanol (50–100%). Subsequently, ethanol was removed with xylene, and the samples were embedded in paraffin wax. Overall, 8 μm thick sections of embedded samples were cut using a Leica RM2235 microtome (Leica, Wetzlar, Germany). The sections were deparaffinized, rehydrated, and stained with 0.1% toluidine blue. The starch granules were individually stained with fuchsin basic. The stained sections were observed using a light microscope (Olympus BX51).

### Transmission electron microscopy

WT and mutant kernels were collected from the same ear of self-pollinated ears from the *dek66* heterozygotes at 15 days after pollination (DAP). The endosperm was cut into small pieces, fixed with 2.5% paraformaldehyde, postfixed with 2.5% (w/v) osmium tetraoxide, dehydrated, and embedded in epoxy resin. Ultrathin sections of 80 nm thickness were cut using a diamond knife, stained with uranyl acetate and lead citrate, and photographed using a transmission electron microscope (JEM-1400Plus; JEOL) at Shandong Agricultural University.

### Embryo rescue *in vitro*

For embryo rescue experiments, embryos were excised at 20 DAP from self-pollinated segregating ears and cultivated in dark at 22 °C on 1/2 Murashige and Skoog salts medium with 6% glucose and 0.3% agar.

### Measurement of proteins, starch, soluble sugars, and C/N

In total, 60 mature kernels of the WT and mutants from three individual self-pollinated ears in the *dek66* heterozygotes were collected and soaked in water for 10 min. Further, the pericarp and embryo were removed, and the endosperm was dried at 37 °C until a constant weight was obtained. The dried endosperm was ground into fine powder using steel beads for subsequent analysis.

The zein, non-zein, and total proteins were extracted from 50 mg dried endosperm powder using sodium borate buffer (12.5 mM sodium borate, 1% SDS, 1 mM phenylmethylsulfonyl fluoride, and 2% 2-mercaptoethanol) as described previously (Chen *et al*., 2013). Protein content was quantified using the Detergent Compatible Bradford Protein Assay Kit (Beyotime, cat. no. P0006C) as per the manufacturer’s instructions.

For starch content measurement, 50 mg dried endosperm powder was mixed with 80% ethanol and heated at 80 °C for 40 min to remove the soluble sugar in the endosperm. The suspension was centrifuged at 11 000 *g* for 10 min. The precipitate was resuspended in 1.2 M sodium acetate buffer and hydrolysed with amyloglucosidase at 50 °C. The glucose produced was quantified by treating with a glucose oxidase/peroxidase (GOPOD, Megazyme) reagent and measuring the absorbance at 510 nm using a spectrophotometer.

The content of soluble sugar was determined using a plant soluble sugar assay kit (Solarbio) as per the manufacturer’s instructions. Using 4 mg dried endosperm powder, the total C/N ratio was measured on an automated Makro N Analyzer with the recommended settings at Shandong Agricultural University.

### Map-based cloning of DEK66

To identify the causative gene for *dek66* phenotype, the F_2_ segregating population was derived from a cross between *dek66/+* and the maize inbred line S162. The *DEK66* locus was mapped using 1652 homozygous mutant kernels. Preliminary mapping was performed using approximately 100 polymorphic molecular markers distributed throughout the maize chromosome. For fine mapping, additional molecular markers (Supplementary Table S1) were developed to narrow the *DEK66* locus to a 66-kb region that contained five annotated genes. To identify the mutation site, the candidate genes were amplified from mutant and WT kernels using KOD DNA polymerase (Toyobo) and were sequenced.

### Vector construction for maize transformation

We performed CRISPR/Cas9-based gene editing of *DEK66* with pBUE411 backbone (Xing *et al.*, 2014). Designed primers with two specific target sites (Supplementary Table S1) and the pCBC-MT1T2 plasmid were subjected to PCR. The PCR fragment and the binary vector pBUE411 were used to set up restriction–ligation reactions using *Bsa*I and T4 ligase, resulting in an expression construct *DEK66*-Cas9-pBUE411 that contained two desired gRNAs.

The *DEK66*-Cas9-pBUE411 expression construct was transformed into the maize B104 inbred line using *Agrobacterium*-mediated transformation at Shandong Agricultural University. T_0_ transformants were screened to identify CRISPR/Cas9-induced mutations in the targeted genes. Further, these mutant transformants were upscaled and screened in F_2_ to obtain CRISPR/Cas9-edited plants lacking the *Cas9* gene. To determine the knockout transformants, target sites of *DEK66* were amplified using the primer pair DEK66-Cas9-F/R and sequenced.

To create the complementation vector, the full-length coding region of *DEK66* was cloned into the pCAMBIA3300 vector under control of the Ubiquitin promoter, yielding the construct *Ubi::DEK66*. Sequences of the primers (*DEK66*-His-F/R) used for vector construction and identification of *DEK66* overexpression transgenic lines are listed in Supplementary Table S1.

### Blue native PAGE and determination of mitochondrial complex activity

Crude mitochondria were isolated from the endosperm of *dek66* mutant and WT at 15 DAP using a plant mitochondrial isolation kit (cat. no. P0045, Biohao, Wuhan, China). Blue native (BN)-PAGE and an in-gel activity assay of mitochondrial complexes were performed as previously described (Ren *et al.*, 2020).

### Western blotting

Total protein was extracted from 1 g of ground frozen endosperm with lysis buffer containing 150 mM NaCl, 10 mM Tris–HCl (pH 7.5), 0.5% NP-40, 2 mM EDTA, and 0.1% protease inhibitor. Western blotting was performed according to the method given previously (Chen *et al.*, 2019). Loading samples were prepared from crude mitochondria or total protein. Primary antibodies used were those against maize cytochrome *c*_1_ (Cyt_*c*1_, 1:5000), alternative oxidase (AOX, 1:10 000), and wheat NADH dehydrogenase subunit 9 (Nad9, 1:3000; a gift from Dr Baocai Tan, Shandong University, China), Rpl16 (1:1000, Agrisera), Nad3 (1:1000, Agrisera) and Arabidopsis cytochrome *c* oxidase subunit 2 (Cox2; 1:1000, Agrisera), the DEK66 antibodies were prepared by Shandong Sibainuo Biotechnology (Ji’nan).

### Subcellular localization of DEK66

The coding DNA sequence of *DEK66* gene lacking the stop codon was amplified with primers listed in Supplementary Table S1 (*DEK66*-PM999-F and *DEK66*-PM999-R). The amplified sequence was introduced into the vector PM999 by restriction enzymes and recombinase to generate a *p35S::DEK66*-eGFP (enhanced green fluorescent protein) fusion construct. Further, the construct and PM999 blank vector were transformed into maize protoplasts by polyethylene-glycol-mediated transformation as previously described (Wei *et al.*, 2021). Before imaging, the protoplasts were stained with 500 nM mitochondrion-specific dye (MitoTracker Red CMXRos, Thermo Fisher Scientific, Waltham, MA, USA) for 30 min at 37 °C. DEK66–GFP and MitoTracker Red CMXRos were excited at 488 and 561 nm, and emissions were collected at 505–550 nm and 575–615 nm, respectively. Fluorescence was observed using a confocal laser scanning microscope (Zeiss LSM880).

### Recombinant DEK66 protein expression, purification, and GTPase activity assay

The open reading frame of *DEK66* was fused with His in the pET-28a expression vector and expressed in *E. coli* BL21 RIL strain. Cells were grown in Luria-Bertani medium until a density of OD 600 of 0.6–0.8, and 0.2 mM isopropyl β-d-1-thiogalactopyranoside was added. After 16 h induction at 16 ˚C, the isolation of the His fusion DEK66 protein was performed with Ni sepharose from GE Healthcare. GTPase activity of the recombinant DEK66 was measured using the QuantiChrom GTPase Assay Kit (BioAssay Systems) according to the manufacturer’s instructions.

### RNA extraction and reverse-transcription quantitative PCR

Total RNA from each sample was extracted using the Ultrapure RNA Kit (cwbiotech, CW0581M). For each sample, 2 μg of total RNA was subjected to reverse transcription using the FastQuant RT Kit (Tiangen) according to the manufacturer’s instructions. Real-time PCR was performed with three biological replicates using SYBR Green qRT-PCR kit (Tiangen) according to the manufacturer’s instructions on a Light Cycler 96 (Roche Diagnostics). The maize *Actin* gene (Zm00001d010159) was used as the internal control. All primers used in this experiment are listed in Supplementary Table S1.

### Detection of H_2_O_2_ and superoxide anions (O^2−^) using 3,3ʹ-diaminobenzidine and nitroblue tetrazolium staining

H_2_O_2_ and superoxide anions (O^2−^) were detected *in situ* using 3,3ʹ-diaminobenzidine (DAB) and nitroblue tetrazolium (NBT) staining as previously described (Ren *et al.*, 2019). Briefly, WT and *dek66* mutant kernels isolated from self-pollinated segregating ears at 15 DAP were longitudinally cut and immersed in 1 mg ml^−1^ DAB (Sigma-Aldrich) in 50 mM Tris–HCl buffer (pH 5.0) at room temperature in dark for 12 h to detect hydrogen peroxide (H_2_O_2_). NBT staining was performed to detect O^2−^. Briefly, the kernels were immersed in 0.5 mg ml^−1^ NBT solution in 10 mM potassium phosphate buffer (pH 7.6) and incubated in complete darkness at room temperature for 20 min. The images were taken using an Olympus DP72 microscope.

### Terminal deoxynucleotidyl transferase dUTP nick end labeling assay

A terminal deoxynucleotidyl transferase dUTP nick end labeling (TUNEL) assay was performed as described previously with a Fluorescein In Situ Cell Death Detection Kit (Roche) according to the manufacturer’s instructions (Ren *et al.*, 2019).

### Polysome profiling

Polysome profiling was performed as described previously with minor adjustment (Xiong *et al.*, 2020). In brief, polysome was extracted from 1 g of ground frozen endosperm with polysome extraction buffer (0.2 M Tris–HCl, 50 mM KCl, 25 mM MgCl_2_, 50 μg ml^−1^ CHX, 50 μg ml^−1^ CRD, 400 U ml^−1^ RNase inhibitor). Crude ribosomes were subjected to a 10–60% sucrose gradient and spun in a Hitachi CP100WX/P40ST-2068 at 65 000 *g* for 16 h at 4 °C. Fractions containing the 40S, 60S, and 80S ribosomal subunits and polysomes were identified by peaks in absorbance at 254 nm.

## Results

### Defective kernel production by the *dek66* mutant

**Fig. 1.**
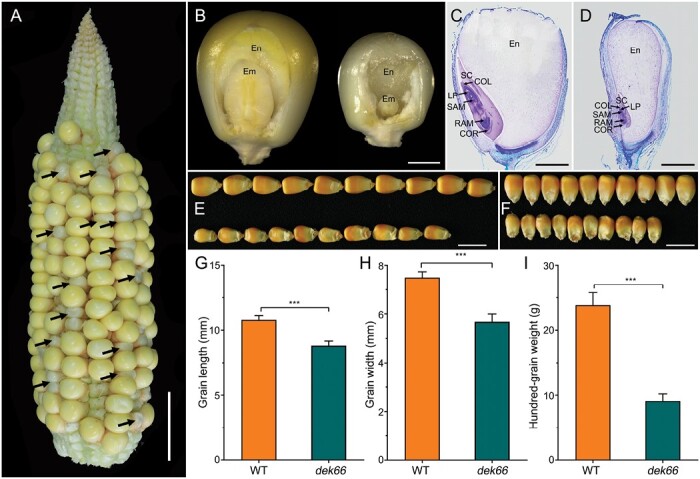
Phenotypes of the *dek66* mutant kernels. (A) A self-pollinated ear at 15 DAP segregates *dek66* mutant kernels (indicated by arrows). Scale bars, 2 cm. (B) Section of WT (left) and *dek66* (right) kernels at 15 DAP. Scale bars, 2 mm. (C, D) Paraffin section analysis of WT (C) and *dek66* (D) kernels at 21 DAP. Scale bars, 2 mm. COL, coleoptile; COR, coleorhiza; LP, leaf primordia; Em, embryo; En, endosperm; RAM, root apical meristem; SAM, shoot apical meristem; SC, scutellum. (E, F) WT (upper) and *dek66* (lower) mature kernels randomly selected from self-pollinated *dek66* heterozygotes ear. Scale bars, 1 cm. (G–I) Comparison of the length, width, and hundred-grain weight of randomly selected mature WT and *dek66* kernels. Error bars indicate the standard deviation (SD). ***P<0.001, Student’s *t*-test.

The *dek66* mutant of maize is an embryo-lethal mutant isolated from an EMS-mutagenized B73 F_2_ population and characterized after backcrosses to B73 and S162 inbred line. The self-pollinated ears of B73/*dek66* and S162/*dek66* heterozygotes contained normal and defective kernels at a ratio 3:1 (Supplementary Table S2), indicating that it was a recessive and nuclear monogenic mutant. The *dek66* mutant kernels were clearly distinguished as smaller and whiter than normal kernels at 15 days after pollination (DAP) (Fig. 1A). Further dissection of WT and *dek66* kernels revealed that the mutant endosperm was translucent, and the mutant embryos were significantly smaller than WT embryos (Fig. 1B). In addition to the significant reduction of embryo size and retardation of leaf primordium, *dek66* embryos established the typical embryonic structure including coleoptiles, shoot apical meristem, coleorhiza, and root apical meristem similar to the WT (Fig. 1C, D). At maturity, the length and width of the *dek66* mutant kernel were only 81.8% and 75.7% those of WT, respectively (Fig. 1E–H), and the hundred-kernel weight of the mutant was nearly 61.8% less than that of WT kernels (Fig. 1I). These results indicated a strong impact of *dek66* mutation on kernel development.

To investigate the developmental status of *dek66* mutants, histological sections of immature mutant and WT kernel from a segregating ear at 9, 12, 15, 18, and 21 DAP were analysed. At as early as 9 DAP, the mutant kernels could be clearly distinguished from WT because of their small size and white color. The endosperm and embryo of the *dek66* mutant were significantly retarded compared with those of WT (Supplementary Fig. S1). Although the embryos of both WT and mutant could establish the typical embryonic structure, the development of mutant endosperm was severely abnormal. It mainly produced BETLs exhibiting structurally irregular cells and cuboidal cell shape; the AL cells were disordered; the central starchy endosperm cells were retarded, and starch accumulation was reduced (Supplementary Figs S2, S3). At 9 DAP, the WT embryo had developed a visible scutellum and shoot apical meristem. In contrast, the *dek66* embryo had only a small scutellum at 15 DAP. At 24 DAP, the WT had developed complete structures of a mature embryo, including leaf primordia, a shoot apical meristem, and a clearly visible root apical meristem. At the same time, the *dek66* mutant also had a complete embryonic structure, but the embryo size was significantly smaller than that of the WT (Supplementary Fig. S3). Considering the size reduction and abnormal embryo of the mutant kernels, we tested their viability using 2,3,5-triphenyltetrazolium chloride staining. Compared with the embryo of WT, the activity of the mutant embryo was considerable reduced, which partially explained why *dek66* embryos could not germinate (Supplementary Fig. S4A–D). To rule out limited nutrition for kernel germination, we cultured WT and *dek66* embryos at 24 DAP from the same self-pollinated *dek66* heterozygote ears on MS medium and observed that *dek66* embryos rescued *in vitro* were retarded in their growth and eventually died (Supplementary Fig. S5). These results indicated that the *dek66* mutation played a role not only in endosperm nutrition accumulation but also in embryogenesis.

### Less protein and starch accumulation in the *dek66* mutant

**Fig. 2.**
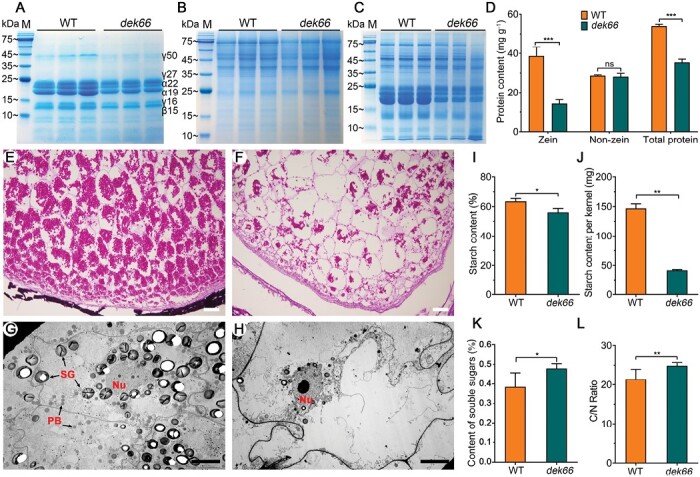
Cytological and biochemical analysis of WT and *dek66* endosperm. (A–C) SDS-PAGE analysis of zein (A), non-zein (B), and total proteins (C) in WT and *dek66*. (D) Quantification of zein, non-zein, and total protein contents in WT and *dek66* mature endosperm. (E, F) Paraffin section analysis of the starch granules in WT (E) and *dek66* (F) kernels at 15 DAP. Scale bars, 500 μm. (G, H) Ultrastructure of the 15 DAP endosperms of the WT (G) and *dek66* (H). Nu, nucleus; PB, protein body; SG, starch granule. Scale bars, 10 μm. (I, J) Quantification of the starch content in WT and *dek66* endosperm at mature stage as reflected by content per weight and content per kernel. (K) The content of soluble sugars in WT and *dek66* endosperm at 15 DAP. (L) The carbon/nitrogen (C/N) ratio in mature WT and *dek66* endosperm. Error bars indicate SD (*n*=3). *P<0.05, ***P<0.001; ns, no significant difference; Student’s *t*-test.

Zein proteins extracted from the WT and *dek66* endosperms were analysed using SDS-PAGE. In *dek66* mutant endosperm, the accumulation of the main storage protein, zein, was decreased compared with that in WT endosperm; however, the content of non-zein proteins was not altered. This resulted in a marked decrease in the total protein content (Fig. 2A–C). This result was confirmed by quantitative measurement of protein content (Fig. 2D). Cytological observation revealed that the accumulation of starch was low in the *dek66* mutant compared with that in WT at 15 DAP (Fig. 2E–H). *dek66* mutant endosperm had substantially lower starch content than WT endosperm, as reflected by the kernel weight (%) and average starch content per kernel (Fig. 2I, J). In contrast, the content of soluble sugar increased in *dek66* mutant endosperm compared with that in WT (Fig. 2K). Furthermore, the C/N ratio of *dek66* mutant endosperm was increased compared with that of the WT (Fig. 2L). These results demonstrated that reduced starch content and insufficient endosperm filling are responsible for the decreased kernel size of the *dek66* mutant.

### Positional cloning of *DEK66*

**Fig. 3.**
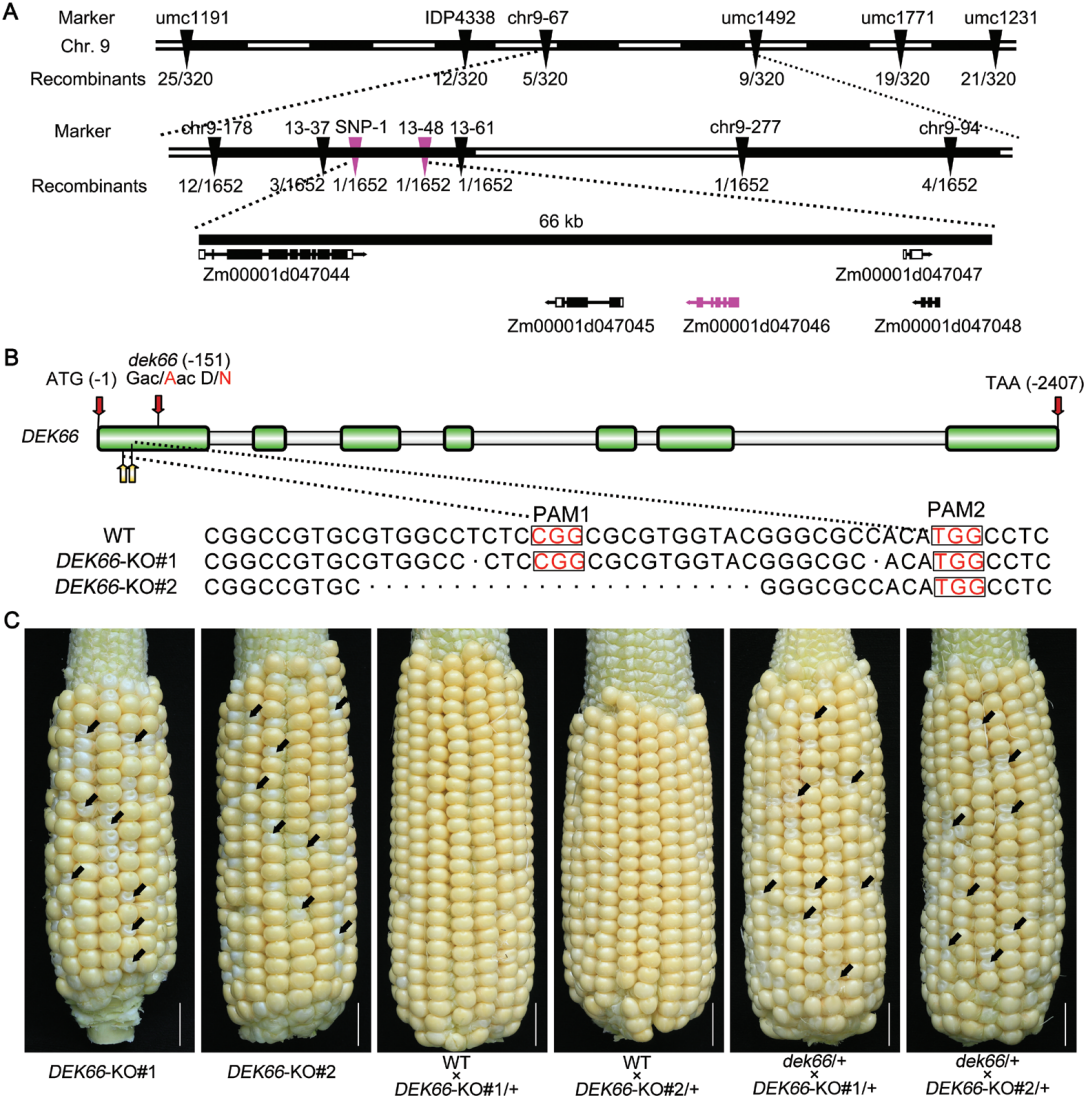
Molecular cloning of *dek66* and CRISPR/cas9-based mutation of *DEK66* and allelism test with *dek66.* (A) Fine-mapping of *dek66* using the F_2_ populations of S162 × *dek66* with 1652 individuals. Between the two markers SNP-1 and 13-48 on chromosome 9, five genes were annotated. (B) The sequence in the Zm00001d047046 locus targeted using CRISPR/Cas9. The protospacer-adjacent motif (PAM) is shown in red letters. Alignments of mutant sequences from two independent transgenic plants are indicated. The broken lines represent deletions. (C) The self-pollinated ear of heterozygous *DEK66*-KO#1 and *DEK66*- KO#2, and allelism test of ear produced from cross heterozygous *DEK66*-KO#1 and *DEK66*- KO#2 to WT and heterozygous *dek66* (*dek66*/+), respectively. Arrows indicate some mutant-phenotype kernels. Scale bars, 2 cm.

To clone the *DEK66* gene, we performed positional cloning using the (S162 × *dek66*) F_2_ population. After characterizing an F_2_ population with a total of 1652 individuals using molecular markers (Supplementary Table S1), the *DEK66* locus was delimited to a 66-kb genomic interval between the markers SNP-1 and 13-48 on chromosome 9, which contained five predicted genes based on the B73 reference genome (RefGen V4) (Fig. 3A). All candidate genes in the *dek66* mutant and WT kernels were amplified and sequenced. Only one single nucleotide mutation (G to A) occurred in the gene Zm00001d047046 in the *dek66* mutant, resulting in GAC (Asp/D) to AAC (Asn/N) codon change (Fig. 3B).

To further confirm that this mutation is responsible for the defective kernel phenotype, we produced two Zm00001d047046-knockout mutant alleles, designated as *DEK66*-KO#1 and *DEK66*-KO#2, respectively, using the CRISPR/Cas9 editing system (Fig. 3B). Genetic analysis indicated that *DEK66*-KO#1 and *DEK66*-KO#2 heterozygote self-pollinated ears contained normal and defective kernels at a ratio 3:1, which was consistent with that of *dek66* (Supplementary Table S2). We performed allelism tests by crossing two independent knockout alleles (*DEK66*-KO#1 and *DEK66*-KO#2) to WT and *dek66* heterozygotes, respectively (Fig. 3C). The results of the allelism test indicated that the kernels displayed a mutant phenotype similar to that of the *dek66* mutant, which confirmed that the *dek66* phenotype is caused by mutation of the Zm00001d047046 gene (Fig. 3C). Additionally, a transgenic functional complementation test was conducted using the coding sequence DNA fragment of Zm00001d047046, driven by the maize *Ubiquitin* promoter. Three independent transgenic lines (OE-1, OE-2, and OE-3) were obtained and confirmed by western blotting and expression analysis (Supplementary Fig. S6A, B). We crossed *DEK66* transgenic lines (OE-1) with the *dek66* (+/−) heterozygous plants and selfed the F_1_ to obtain the F_2_ progeny, and then the F_2_ kernels with WT and mutant phenotype were genotyped by PCR (Supplementary Fig. S6C, D). The Indel-1F/R markers were used to detect the *dek66* locus of F_2_ kernels, and the genotype of B73 and CK represent the mutant and WT, respectively. Genotyping 24 of the F_2_ kernels identified three kernels (numbered 2, 5, and 7) that were homozygous for *dek66* harboring the *DEK66* transgene, and these three kernels showed a normal phenotype compared with the WT. In contrast, the kernels numbered 13–24 contain a homozygous *dek66* locus without the *DEK66* transgene and show the mutant phenotype (Supplementary Fig. S6D–H). The results indicate that overexpression of *DEK66* rescued the defective phenotype of this mutant, which validated Zm00001d047046 as the site of the *dek66* mutation.

To assess whether the enhanced expression of *DEK66* could increase grain yield, kernel traits were examined using T_2_ generations of *DEK66* overexpression transgene lines (OE-1, OE-2, and OE-3) and CK. As shown in Supplementary Fig. S6I–M, OE-1, OE-2, and OE-3 have no significant difference from CK in the kernel size and hundred-kernel weight.

### Mitochondrion-targeted GTPase encoded by *DEK66*

**Fig. 4.**
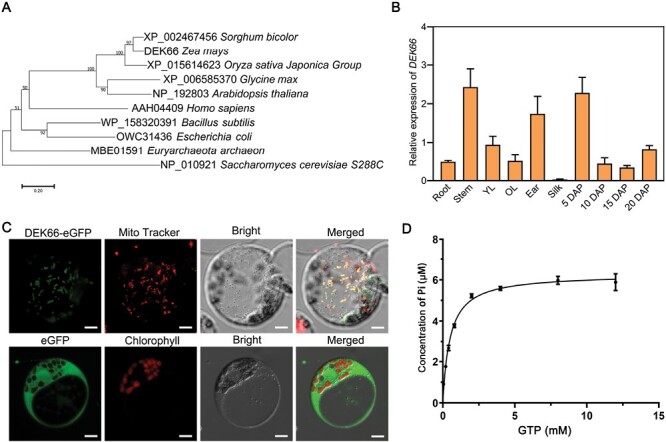
Phylogenetic analysis and expression pattern of *DEK66*, and subcellular localization of DEK66. (A) Phylogenetic relationship of DEK66 homologs from representative species. Bar, 0.05 substitutions per site. (B) Expression pattern analysis of *DEK66* by qRT-PCR. Normalization was performed against the maize *Actin* gene (Zm00001d010159). OL, old leaves; YL, young leaves. Values represent the mean and SD of three biological replicates; the developing kernels are at 5, 10, 15, and 20 DAP. (C) DEK66–eGFP fusion protein and free eGFP were transiently expressed in protoplasts of three-leaf-stage etiolated maize seedlings. Mitochondria are marked by MitoTracker (red). Scale bars, 10 μm. (D) Kinetic analysis of GTP hydrolysis rates of DEK66 proteins.

To further investigate the evolutionary history of DEK66, we built a phylogenetic tree of DEK66 of various species with the full-length protein sequence of DEK66 and its putative ortholog via BLAST in the GenBank database. The phylogenetic tree and alignment suggested that DEK66 and other proteins in the plant species had a close evolutionary relationship (Fig. 4A). A detailed sequence alignment among various species indicated that DEK66 harbored the conserved GTP-binding domain, G4–G1–G2–G3, and DAR and LxG domains (Supplementary Fig. S7). *DEK66* was constitutively expressed in all detected tissues, and its highest transcript level was detected in the kernels at the early developmental stage (Fig. 4B). To determine the subcellular localization of DEK66, transient expression experiments were conducted in maize mesophyll cells using a fused construct, *p35S::DEK66*-eGFP. It was demonstrated that GFP signal co-localized with MitoTracker Red (a mitochondrial probe), indicating a mitochondrial principal localization of DEK66 (Fig. 4C). This suggested that *DEK66* may encode a GTPase that is a member of the ribosome biogenesis GTPase A (RbgA) family.

To assess the activity of DEK66, we expressed the recombinant DEK66-His in *Escherichia coli* and measured the GTP hydrolysis activity *in vitro* using the QuantiChrom GTPase Assay Kit. Further, we determined dependence of the GTPase activity on substrate concentration by incubating 2 μg DEK66-His with varied concentrations of GTP (0–15 mM). The results revealed that after hydrolysis in the presence of a certain amount of DEK66-His protein, the concentration of free P_i_ produced increased with substrate (GTP) concentration. However, when the substrate concentration reached 4 mM, the concentration of free P_i_ produced by hydrolysis did not continue to increase because of the restriction of the amount of DEK66-His protein (Fig. 4D). This indicated that DEK66 has GTP hydrolase activity.

### Steady-state level analysis of mitochondrial transcripts and proteins

*DEK66* encodes a mitochondrion-targeted GTPase, a member of the RbgA family, which is reported to be mainly involved in ribosomal assembly in prokaryotes and eukaryotes (Britton, 2009; Jeon *et al.*, 2017). To verify whether DEK66 is involved in the regulation of mitochondrial ribosome function, the transcript levels of ribosomal-protein-encoding genes in mitochondria were determined. Such genes were up-regulated to varying degrees in *dek66* mutant endosperm cells (Supplementary Fig. S8). This indicated that the *DEK66* mutation affects the function of mitochondrial ribosomes to a certain extent.

**Fig. 5.**
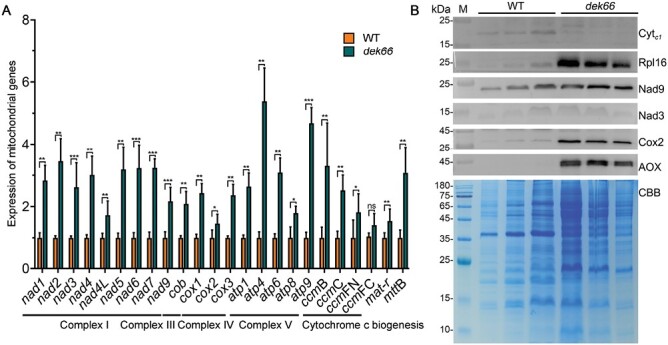
Steady-state level analysis of mitochondrial transcripts and proteins in WT and *dek66* endosperm. (A) Mitochondrial gene expression analysis by qRT-PCR. Normalization was performed against the maize *Actin* gene (Zm00001d010159). Values represent the mean and SD of biological replicates (*n*=3), *P<0.05, **P<0.01, ***P<0.001; ns, no significant difference; Student’s *t*-test. (B) Steady-state level analysis of mitochondrial proteins in WT and *dek66* endosperm. CBB, Coomassie brilliant blue.

The maize mitochondrial genome is reported to have 35 protein-coding genes, including 22, 11, 1, and 1 genes encoding electron transport chain proteins, ribosomal proteins, a maturase (*mat-r*), and a transporter protein (*mttB*), respectively (Clifton *et al.*, 2004). To investigate the function of DEK66, we further compared the transcript levels of mitochondrial genes in the WT and *dek66* endosperms at 15 DAP. Most mitochondrial genes were up-regulated in the *dek66* mutant, indicating that the loss of function of DEK66 affected the transcription of mitochondrial genes (Fig. 5A). To further investigate the levels of proteins in the mitochondria, we isolated mitochondrial proteins from the WT and *dek66* endosperms at 15 DAP and quantified them using western blotting. Mitochondrial proteins were up- or down-regulated to varying degrees in the *dek66* mutant (Fig. 5B). Collectively, our results indicated that DEK66 affects mitochondrial gene transcription and translation.

### Effect of *DEK66* on mitochondrial function and morphology

**Fig. 6.**
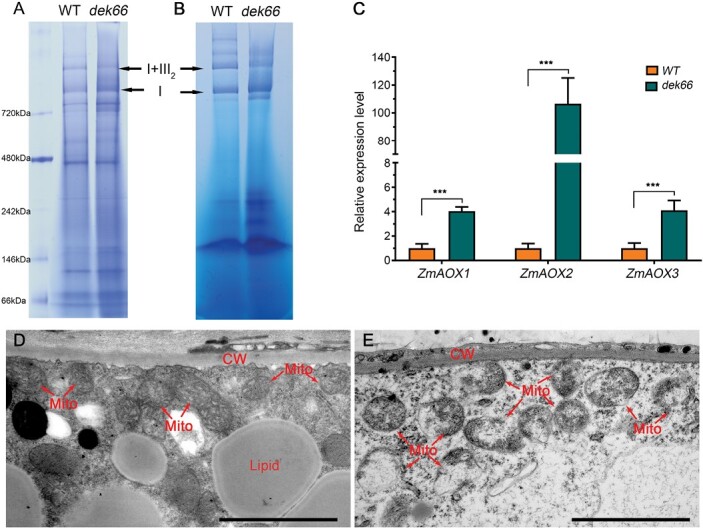
Mitochondrial function affected in *dek66* mutant seeds. (A) BN-PAGE analysis of mitochondrial complexes. The positions of super-complex I+III_2_ and complex I are indicated. (B) In-gel NADH dehydrogenase activity test of complex I. The positions of complex I and super-complex I+III_2_ are indicated. (C) Alternate oxidase gene (*ZmAOX1*, *ZmAOX2*, *ZmAOX3*) expression analysis of *dek66* by qRT-PCR. Normalization was performed against the maize *Actin* gene (Zm00001d010159). Values represent the mean and SD of three biological replicates. ***P<0.001, Student’s *t*-test. (D, E) Ultrastructure of developing endosperms from WT and *dek66* seeds (15 DAP) for mitochondria observation. Scale bars, 2 mm. CW, cell wall; Mito, mitochondrion.

To determine whether the mitochondrial complexes are affected in the *dek66* mutant, the crude mitochondrial proteins were isolated from WT and *dek66* endosperms at 15 DAP, and the protein complexes were separated using native PAGE. Coomassie brilliant blue staining and in-gel NBT NADH activity staining revealed that the abundance and activity of super-complex CI+CIII_2_ were dramatically decreased in the *dek66* mutant (Fig. 6A, B). A deficiency in the cytochrome respiratory pathway usually leads to activation of the alternative oxidase pathway to maintain homeostasis in mitochondria (Kuhn *et al.*, 2015). The transcript levels of *AOX* genes were analysed in WT and the *dek66* mutant. The transcript levels of *ZmAOX1*, *ZmAOX2*, and *ZmAOX3* were significantly up-regulated in the *dek66* mutant by approximately 4-, 106-, and 4-fold, respectively, compared with those in WT. This indicated that *DEK66* mutation inhibits respiration while enhancing the AOX pathway (Fig. 6C).

Normal activation of the electron transport chain is required for the proper formation of the inner envelope cristae in mitochondria (Logan, 2006). To further examine the variation in mitochondrial ultrastructure, WT and *dek66* endosperms at 15 DAP were observed under a transmission electron microscope. Compared with WT, the majority of the mitochondria in the *dek66* mutant were enlarged, deformed, and had void structures, and the cristae formed by the inner membrane were strongly reduced in number (Fig. 6D, E). These results suggest that mitochondrial function was severely disrupted in *dek66* mutant.

### Excessive reactive oxygen species accumulation and severe DNA damage in *dek66* mutant endosperm

**Fig. 7.**
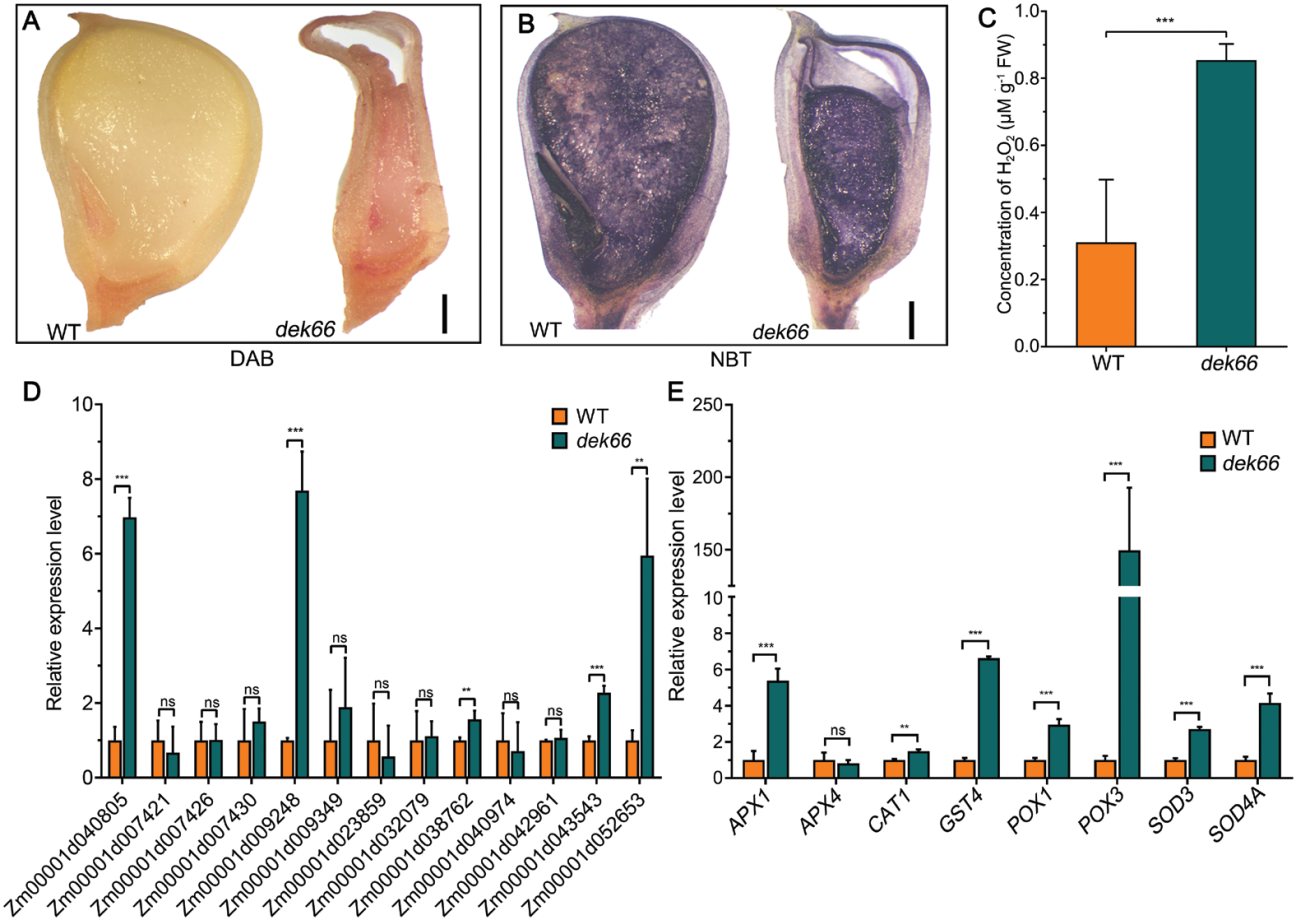
Determination of reactive oxygen species (ROS) contents and expression analysis of genes related to ROS production and detoxification. (A, B) 3,3ʹ-Diaminobenzidine (DAB; A) and nitroblue tetrazolium (NBT; B) staining for the detection of hydrogen peroxide (H_2_O_2_) and superoxide (O^2−^) in endosperm of the WT and *dek66* at 15 DAP. Scale bars, 1 mm. (C) Concentration of H_2_O_2_ in 15 DAP endosperm of WT and *dek66*. (D) Expression analysis of NADPH oxidase family genes in 15 DAP endosperm of WT and *dek66*. (E) Expression analysis of antioxidant-related genes in 15 DAP endosperm of WT and *dek66*. *AOX2*, alternative oxidase 2; *APX4*, ascorbate peroxidase 4; *CAT1*, catalase 1; *GST4*, glutathione-*S*-transferase 4; *POX1*, guaiacol peroxidase 1; *POX3*, guaiacol peroxidase 3; *SOD3*, superoxide dismutase 3; *SOD4A*, superoxide dismutase 4A. Values are means with SD (*P<0.05, **P<0.01, ***P<0.001; ns, no significant difference; Student’s *t*-test).

Reactive oxygen species (ROS) are mainly produced by mitochondrial electron transport chain complexes I and III (Gill and Tuteja, 2010; Sousa *et al.*, 2018). The contents of H_2_O_2_ and O_2_^−^ in WT and *dek66* mutant endosperms were determined using DAB and NBT staining, respectively. *dek66* mutant endosperm accumulated a large amount of ROS (Fig. 7A, B). Corresponding quantitative assays revealed that the H_2_O_2_ levels were significantly higher in *dek66* mutant endosperm than in WT endosperm (Fig. 7C). These results indicated that loss of function of DEK66 resulted in the accumulation of ROS in *dek66* mutant endosperm. ROS production is usually accompanied by enhanced NADPH oxidase activity (Zhang *et al*., 2020). The NADPH oxidase family in maize comprises 13 genes, among which five genes (Zm00001d040805, Zm00001d009248, Zm00001d038762, Zm00001d043543, and Zm00001d052653) were significantly up-regulated in *dek66* mutant endosperm as revealed by qPCR (Fig. 7D). Collectively, these results indicated that the up-regulation of NADPH oxidase genes by *DEK66* mutation leads to increased NADPH oxidase activity in the *dek66* mutant, which in turn leads to excessive ROS accumulation.

Plants have evolved an antioxidant system to scavenge excessive ROS in cells (Mittler, 2017). The expression of these antioxidant enzymes, including ascorbate peroxidase (APX), catalase (CAT), glutathione-*S*-transferase (GST), guaiacol peroxidase (POX), and superoxide dismutase (SOD), is usually increased when ROS levels are elevated (Mittler, 2017). A series of typical ROS-responsive genes encoding these antioxidant components, namely, *APX1*, *CAT1*, *GST4*, *POX1*, *POX3*, and *SOD4A* (Mylona *et al.*, 2007), displayed higher transcript levels in the developing kernels of the *dek66* mutant at 15 DAP (Fig. 7E). This was consistent with the elevated ROS accumulation in the mutant.

**Fig. 8.**
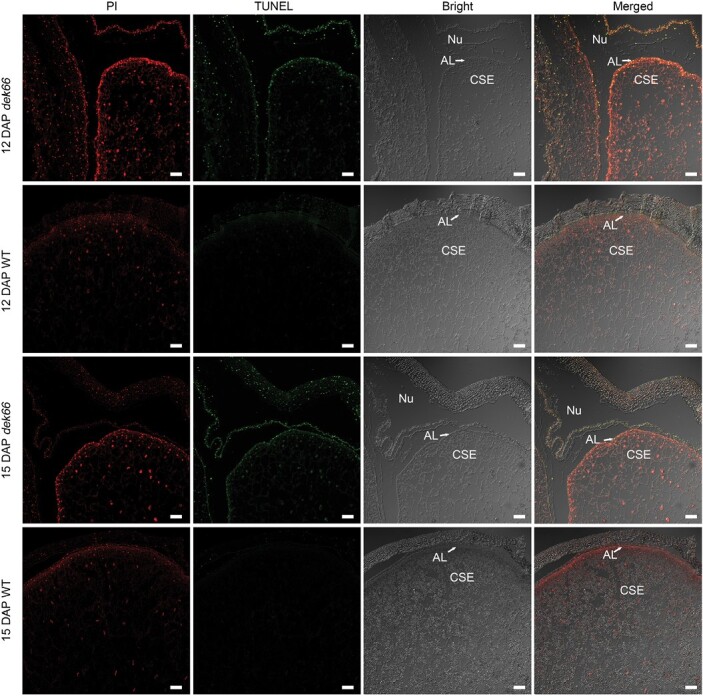
Determination of DNA damage. DNA damage measured by terminal deoxynucleotidyl transferase dUTP nick-end labeling (TUNEL) in 12 DAP and 15 DAP kernels of WT and *dek66*. Red signal is propidium iodide (PI) staining; green color represents positive results. Scale bars, 100 μm. AL, aleurone; NU, nucellus; CSE, central starchy endosperm.

ROS are highly reactive and toxic; their overproduction in plants damages proteins, lipids, carbohydrates, and DNA, ultimately resulting in oxidative stress (Gill and Tuteja, 2010; Ye *et al.*, 2021). To assess the effect of excessive ROS accumulation on genomic DNA, we performed a TUNEL assay on the WT and the *dek66* mutant using longitudinal sections of kernels at 12 and 15 DAP. The same samples were simultaneously stained with propidium iodide to reveal the nuclei (red) in each section. In the cells of WT kernels, very few nuclei were TUNEL-positive (Fig. 8). However, significantly more nuclei of the central starchy endosperm and AL cells of the *dek66* mutant were TUNEL-positive (Fig. 8). Thus, *DEK66* null mutation results in severe DNA damage in the nucellus, central starchy endosperm, and AL cells of *dek66* mutant kernels.

### Modulation of key genes involved in maize kernel development by DEK66

To elucidate the developmental defects of the *dek66* mutant in terms of gene transcript level, we performed RNA sequencing (RNA-seq) using total RNA isolated from WT and *dek66* mutant endosperms at 15 DAP. A total of 2701 differentially expressed genes (DEGs) were identified between WT and the *dek66* mutant, of which 1509 genes were down-regulated and 1201 genes were up-regulated in the *dek66* mutant (Supplementary Fig. S9A). Gene ontology (GO) enrichment analysis of these DEGs revealed that they were mainly significantly enriched in nutrient storage, ATPase activity, DNA replication, cell cycle, and other related biological processes (Supplementary Fig. S9B). At the same time, Kyoto Encyclopedia of Genes and Genomes (KEGG) metabolic pathway cluster analysis of DEGs revealed that they were mainly enriched in biological pathways related to DNA replication and nutrient metabolism (Supplementary Fig. S9C). Interestingly, we observed that most genes involved in the synthesis and metabolism of the nutrient reservoir activity were down-regulated in the endosperm of the *dek66* mutant, which was consistent with the phenotype of reduced starch and protein contents in the mutant. These results indicated that the decrease in starch and protein contents might be due to the down-regulation of the genes involved in starch and protein biosynthesis, to some extent. In addition, the transcript level of many genes related to cell growth, division, cell cycle, and programmed cell death were significantly different between WT and the *dek66* mutant (Supplementary Fig. S9D). Therefore, DEK66 might be involved in nutrient accumulation and metabolism through direct or indirect regulation of key genes during maize kernel development.

*DEK66* encodes a mitochondrial localized GTPase and is involved in regulating mitochondrial ultrastructure and function. To further understand how DEK66 regulates mitochondrial function in terms of gene transcript level, we screened DEGs related to mitochondria from RNA-seq data for further analysis. Among all DEGs, 157 were involved in the regulation of mitochondrial function, among which 47 genes were down-regulated and 110 were up-regulated in the *dek66* mutant (Supplementary Fig. S10A). KEGG pathway cluster analysis revealed that these DEGs were mainly enriched in the tricarboxylic acid cycle, carbon metabolism, pyruvate metabolism, and other pathways related to energy metabolism (Supplementary Fig. S10B).

GO cluster analysis revealed that the genes related to mitochondrial components, mitochondrial membrane, mitochondrial respiratory chain complex, ribosomes, and other processes in cell components were significantly enriched. Genes related to redox, energy metabolism, tricarboxylic acid cycle, and other processes in biological processes were enriched, which may result in defective energy metabolism in the *dek66* mutant. The genes associated with molecular functions including mitochondrial ribosome structural composition, transferase activity, electron transport activity, GTPase activity, and other processes were enriched, indicating that mitochondrial ribosomal assembly and GTPase activity were affected in the *dek66* mutant (Supplementary Fig. S10C). Collectively, our results further confirmed that *DEK66* mutation accompanied by differential expression of key genes was involved in maize kernel development.

## Discussion

### *DEK66* encodes a ribosomal assembly factor located in the mitochondria

Based on the phylogenetic tree constructed from the full-length sequence of DEK66 and potential homologous protein sequences of other species, DEK66 is widely conserved in monocots, dicots, mammals, yeast, bacteria, and archaea and belongs to the RbgA family (Fig. 4A; Supplementary Fig. S7). Recent studies reported the biological function of some ribosomal assembly factors that are putative orthologs of DEK66. *Bacillus subtilis* YlqF appears to have an essential function in the assembly of the 50S subunit (Matsuo *et al.*, 2006; Uicker *et al.*, 2006). Members of the RbgA family are found in all eukaryotes and are implicated in the assembly of cytoplasmic, plastid, and mitochondrial ribosomes. In yeast, the RbgA homolog cytoplasmic GTPase Lsg1 has been proposed to play a role in the incorporation of Rpl10 into the large ribosomal subunit (Hedges *et al.*, 2005). *Nicotiana benthamiana* RbgA (NbRbgA) localized in the chloroplasts possesses GTPase activity and functions in chloroplast rRNA processing/ribosome biogenesis, affecting chloroplast protein translation in higher plants (Jeon *et al.*, 2017). Functional studies on humans and *Saccharomyces* MTG1 proteins manifest their conservative function in ribosomal assembly in mitochondria (Barrientos *et al.*, 2003; Kotani *et al.*, 2013). Collectively, this indicates conservation of a ribosome biogenesis function among divergent RbgA proteins in the subcellular compartments where they are located.

Translation of mitochondrial genes encoding electron transport chain complexes can be differentially affected by alterations in mitochondrial ribosomes (Janska and Kwasniak, 2014). Abnormal expression of mitochondrial genes and proteins was observed in the *dek66* mutant (Fig. 5; Supplementary Fig. S8). In addition, the abundance of 40S ribosomal small subunits was significantly lower in the *dek66* mutant than in WT (Supplementary Fig. S11). The results revealed that the loss of function of DEK66 affected the abundance of small cytosolic ribosomal subunits. It is reported that ribosomal translation in mitochondria and cytoplasm maintains a dynamic balance; when ribosomal translation in mitochondria is inhibited, protein translation in cytoplasm is also inhibited, which is a universal phenomenon from nematodes to mammals (Molenaars *et al.*, 2020). Therefore, we strongly believe that DEK66 is a member of the RbgA family, possesses GTPase activity, and plays a role in mitochondrial ribosomal assembly in maize.

### DEK66 is involved in the regulation of mitochondrial function and kernel development in maize

A surprising outcome from the gene cloning of maize kernel mutants was an overwhelming portion of cloned genes encoding pentatricopeptide repeat (PPR) proteins. As reported, dysfunction of most PPR and some mitochondrial proteins causes impaired assembly and activity of mitochondrial complexes; this results in defective mitochondrial function and morphology, leading to severely altered kernel phenotype (Qi *et al.*, 2019; Fan *et al.*, 2021; Cao *et al.*, 2022; Feng *et al.*, 2022). In this study, we identified a new mitochondrial gene, *DEK66*, which encodes a ribosomal assembly factor. Dysfunction of *DEK66* causes specifically arrested development of maize kernels. Compared with WT kernels, *dek66* mutant kernels exhibited a reduced proportion of hard endosperm and small embryos (Fig. 1). *dek66* mutant embryos had the typical embryonic structure; however, *dek66* mutant seedlings could only be obtained by embryo rescue *in vitro*, were retarded in their growth, and eventually died. DEK66 played a role not only in embryogenesis but also in morphogenesis of seedlings, which is supported by its constitutive expression in maize (Fig. 4B).

The BETL is mainly responsible for transporting nutrients from maternal tissues to endosperm cells of the offspring and is critical for normal kernel development in maize (Offler and Patrick, 2020; Hu *et al.*, 2021; Wang *et al.*, 2022). In this study, the *dek66* mutant had structurally irregular cells in the BETL and failed to develop elongated cell wall ingrowth similar to those of WT, which may lead to less deposition of storage reserves in the endosperm (Supplementary Fig. S2). More importantly, previous studies indicated that many mutants with impaired mitochondrial function exhibit abnormal development of BETL (Zhang *et al.*, 2017; Dai *et al.*, 2018, 2020; Chen *et al.*, 2021; Fan *et al.*, 2021). In addition, many mitochondria in the developing endosperm transfer cells were highly abundant (Davis *et al.*, 1990; Offler *et al.*, 1997; Kang *et al.*, 2009; Monjardino *et al.*, 2013). That is, the presence of a large number of mitochondria probably supplied energy for the synthesis of cell wall ingrowths and for solute exchange in plasma membrane outlining beneath the wall ingrowths during kernel development (Zheng *et al.*, 2015). The loss of function of DEK66 led to abnormal mitochondrial structure and function, which may be the main reason for the morphological abnormality of BETL and kernel development in the *dek66* mutant (Fig. 6). However, the mechanism underlying the regulation of the differentiation of transmission cells and nutrient transport by mitochondria still needs to be further studied.

## Supplementary data

The following supplementary data are available at *JXB* online.

Fig. S1. Developmental analysis of the WT and *dek66* mutant kernels.

Fig. S2. BETL, AL, CSE development is retarded and affected in *dek66* mutant kernels.

Fig. S3. Magnified embryo of WT and *dek66*.

Fig. S4. Embryo viability test and rescue.

Fig. S5. Embryo rescue *in vitro.*

Fig. S6. Functional complementation test of *dek66* and kernel traits of *DEK66* transgenic lines (OE-1, OE-2, and OE-3).

Fig. S7. Alignment of the full-length DEK66 protein sequence and homologous protein sequences from different species.

Fig. S8. The expression of mitoribosome genes.

Fig. S9. *DEK66* regulates genes involved in diverse processes.

Fig. S10. Analysis of mitochondrial related differentially expressed genes.

Fig. S11. Polysome profiling assay with sucrose density gradient.

Table S1. List of primers used in this study.

Table S2. Genetic analysis of the kernels in F_2_ population of the segregating ears.

erad289_suppl_Supplementary_Figures_S1-S11_Tables_S1-S2

## Data Availability

All data supporting the findings of this study are available within the paper and its supplementary data published online. The primary database used for experimental procedures and results are deposited at Dryad Digital Repository (DOI: 10.5061/dryad.rfj6q57g6 (Wei *et al*., 2023).

## Abbreviations

AL: aleurone layer
BETL: basal endosperm transfer layer
DAP: days after pollination
OXPHOS: oxidative phosphorylation
WT: wild-type
