# Supplementary material for: Defective kernel 66 encodes a GTPase essential for kernel development in maize
Source: J Exp Bot. 2023 Jul 25;74(18):5694–708. doi: 10.1093/jxb/erad289 (PMC10540730; doi:10.1093/jxb/erad289)
Supplement: erad289_suppl_Supplementary_Figures_S1-S11_Tables_S1-S2 [file erad289_suppl_supplementary_figures_s1-s11_tables_s1-s2.pdf]

Supplemental Information

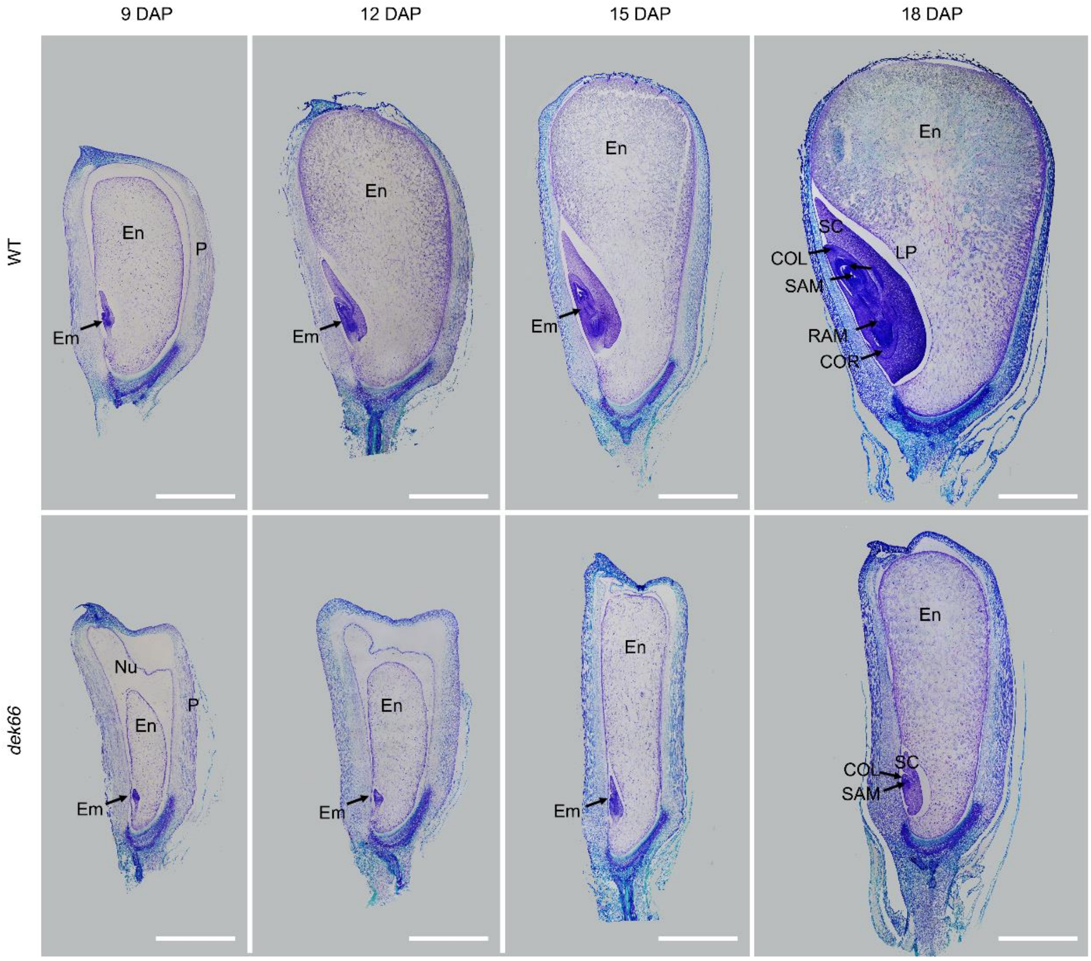

Fig. S1. Developmental Analysis of the WT and *dek66* Mutant Kernels.

Paraffin sections analysis of WT and *dek66* kernels at 9, 12, 15, 18 DAP.

Em, embryo; En, endosperm; Nu, nucellus; LP, leaf primordia; P, pericarp; RAM, root apical meristem; SAM, shoot apical meristem; SC, scutellum; COL, coleoptile; COR, coleorhiza. Scale bars, 2 mm.

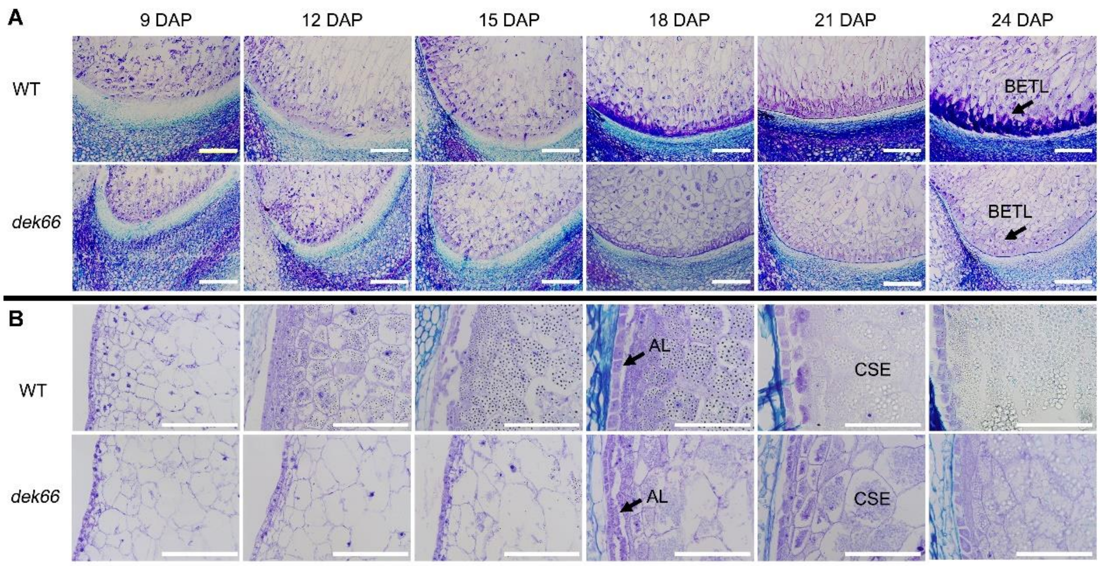

Fig. S2. BETL, AL, CSE Development Is Retarded and Affected in *dek66* Mutant Kernels.

Magnified BETL, AL and CSE of WT and *dek66* kernels at 9, 12, 15, 18, 21, and 24 DAP is indicated by black arrowhead, respectively. Scale bars, 200 μm.

BETLs, basal endosperm transfer cells; AL, aleurone layer; CSE, central starchy endosperm.

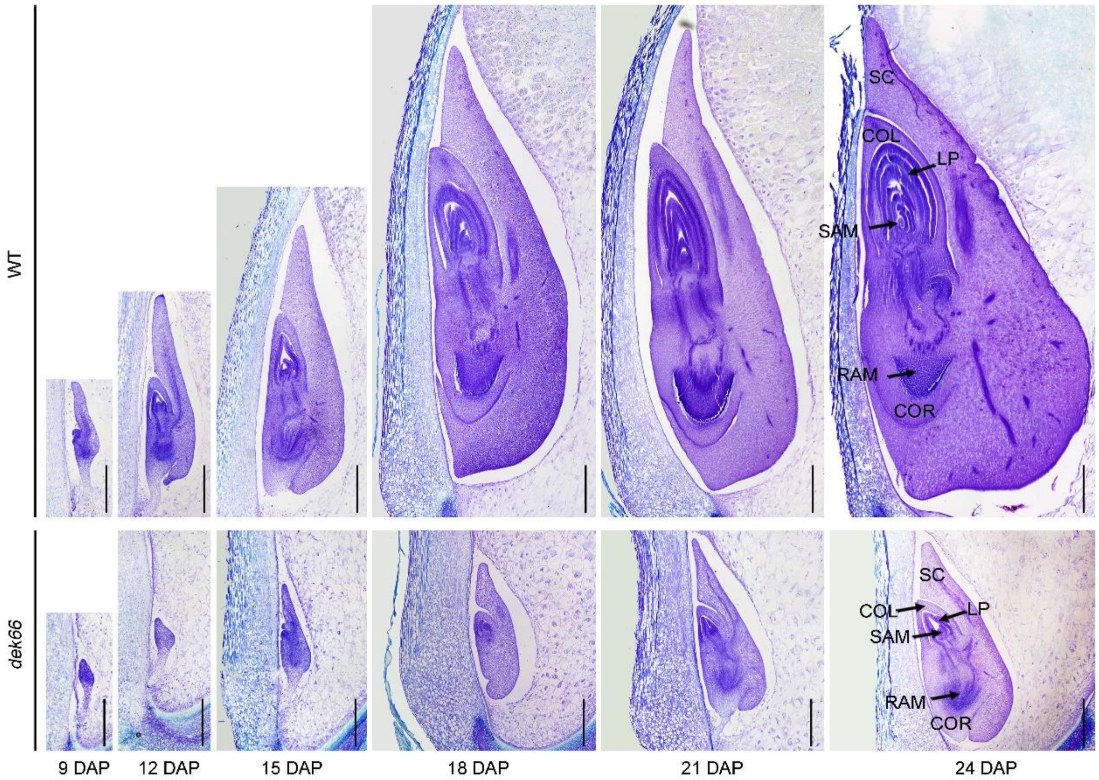

Fig. S3. Magnified embryo of WT and *dek66*.

LP, leaf primordia; P, pericarp; RAM, root apical meristem; SAM, shoot apical meristem; SC, scutellum; COL, coleoptile; COR, coleorhiza. Scale bars, 2 mm.

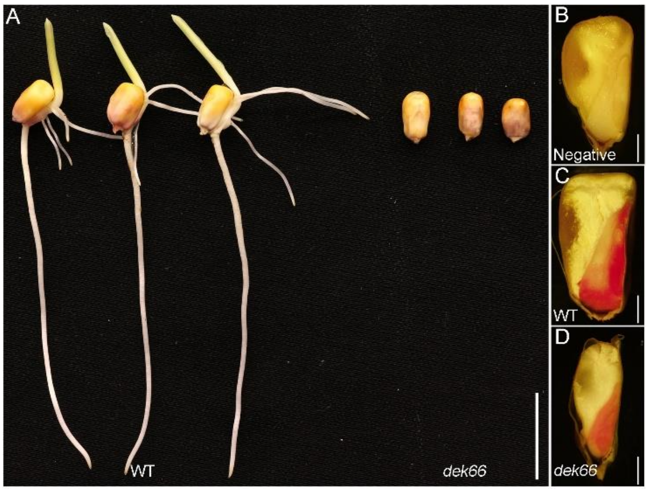

Fig. S4. Embryo viability test and rescue

(A) Seed germination of the WT and *dek66* mutant. Scale bars, 2 cm.

(B-D) Seeds stained with 2,3,5-triphleiiyl-tetrazoliumehloride. Seeds boilled in boiling water for negative control (B).

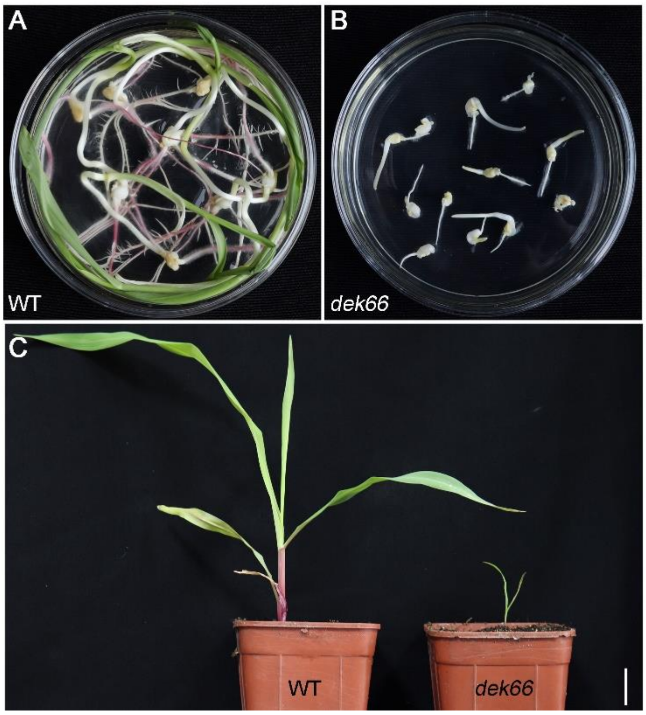

Fig. S5. Embryo rescue in vitro

(A-B) Growth of WT (A) and *dek66* embryos (B) rescued at 24 DAP and cultured on 1/2 MS medium with 6% sucrose. (C) WT (left) and *dek66* seedlings (right) obtained by embryo rescue. Scale bars, 2 cm.

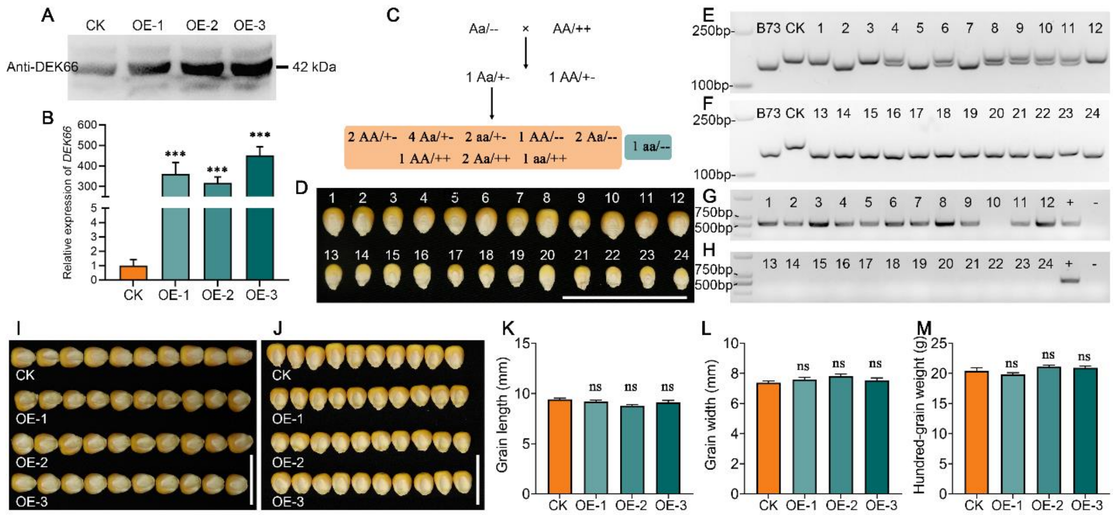

Fig. S6. Functional complementation test of *dek66* and kernel traits of *DEK66* transgenic lines (OE-1, OE-2, and OE-3).

(A) Western blot assay of DEK66 protein level of transgenic lines. CK, non-transgenic control. (B) qPCR assay of *DEK66* mRNA level of transgenic lines. (C) Schematic diagram of *dek66* transgenic complementation. A, *DEK66*; a, *dek66*; +, transgene-transformed *DEK66*; -, untransformed. (D) Phenotypic analysis of transgenic kernels of the complementation test. Representative kernels with the WT (1-12) and mutant (13-24) phenotypes. Scale bars, 2 cm. (E-F) Genotype of the kernels for *dek66* locus using the indel marker (Indel-1F/R). (G-H) The primers of the *DEK66* gene (a forward primer named US5-1 was designed at the 3′ end of maize *Ubiquitin* promoter and the reverse primer named DEK66-R was designed at the coding region of *DEK66* gene) were used to identify the transgenic positive and negative kernels. +, expression vector plasmid as positive control. -, H_2O as negative control. (I-J) CK and *DEK66* transgenic lines (OE-1, OE-2, and OE-3) mature kernels randomly selected from self-pollinated ear. Scale bars, 2 cm. (K-M) Comparison of the length, width, and hundred-grain weight of randomly selected mature kernels. Error bars indicate the standard deviation (SD), ns refers to $P > 0.05$, Student’s test).

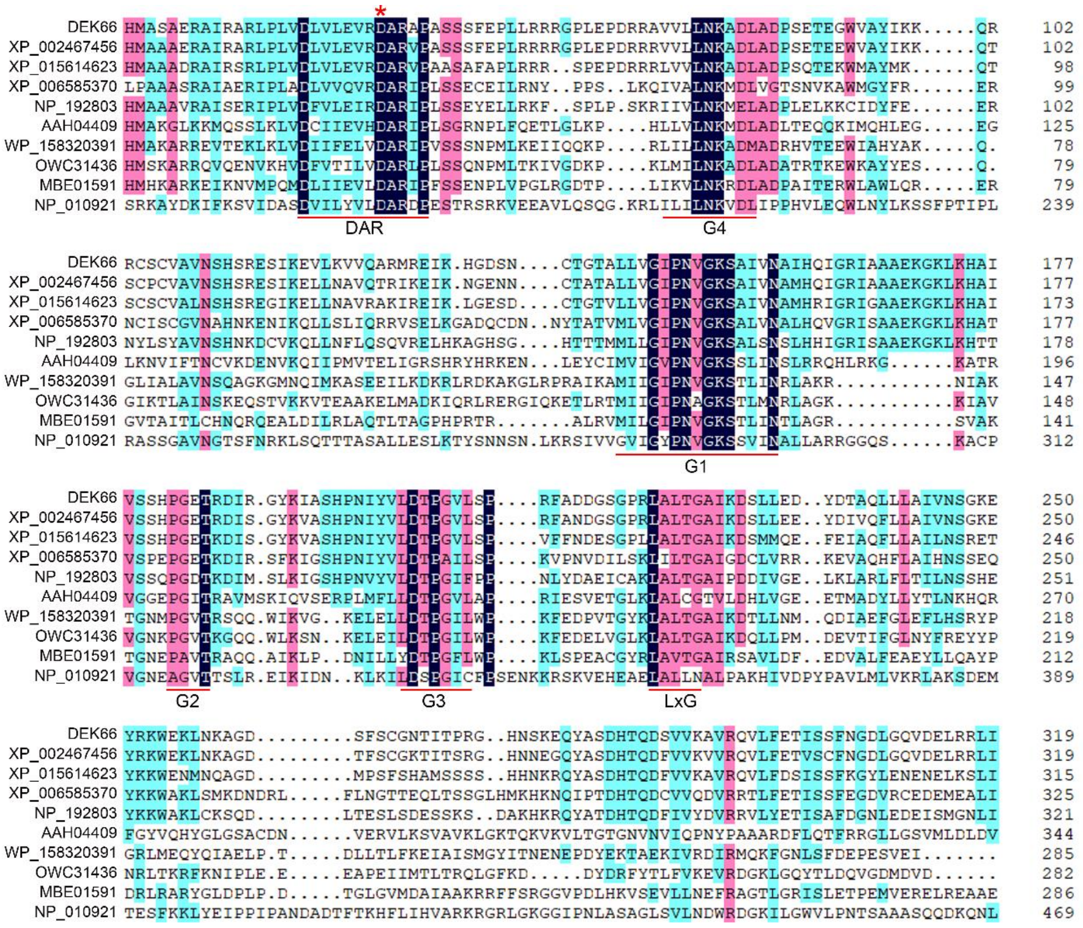

Fig. S7. Alignment of the full-length DEK66 protein sequence and homologous protein sequences from different species

Sorghum bicolor, XP_002467456; Oryza sativa Japonica Group, XP_015614623; Glycine max, XP_006585370; Arabidopsis thaliana, NP_192803; Homo sapiens, AAH04409; Bacillus subtilis, WP_158320391; Escherichia coli, OWC31436; Euryarchaeota archaeon, MBE01591; Saccharomyces cerevisiae S288C, NP_010921. The black regions represent completely conserved residues. The putative DAR motif, GTP binding motif (G4-G1-G2-G3) and LxG motif are underlined and annotated. The mutant site in *dek66* is indicated by red star.

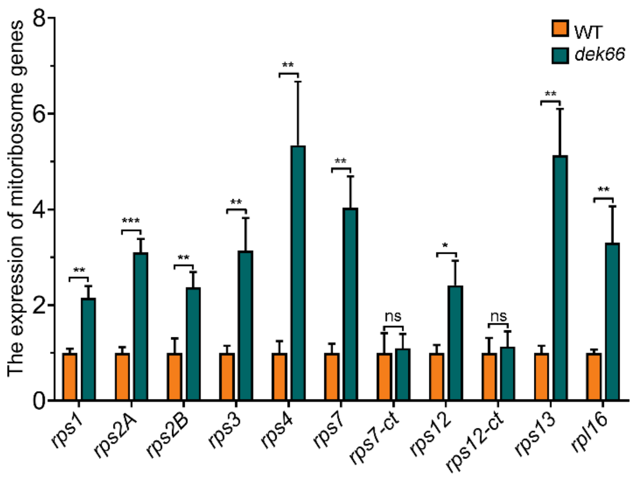

Fig. S8. The expression of mitoribosome genes

Mitoribosome genes expression analysis by qRT-PCR. Normalization was performed against the maize *ZmActin* gene (Zm00001d010159); Values represent the mean and SD of biological replicates (n = 3), * refers to $P < 0.05$, ** refers to $P < 0.01$; *** refers to $P < 0.001$; ns, no significant difference; Student' s *t*-test.

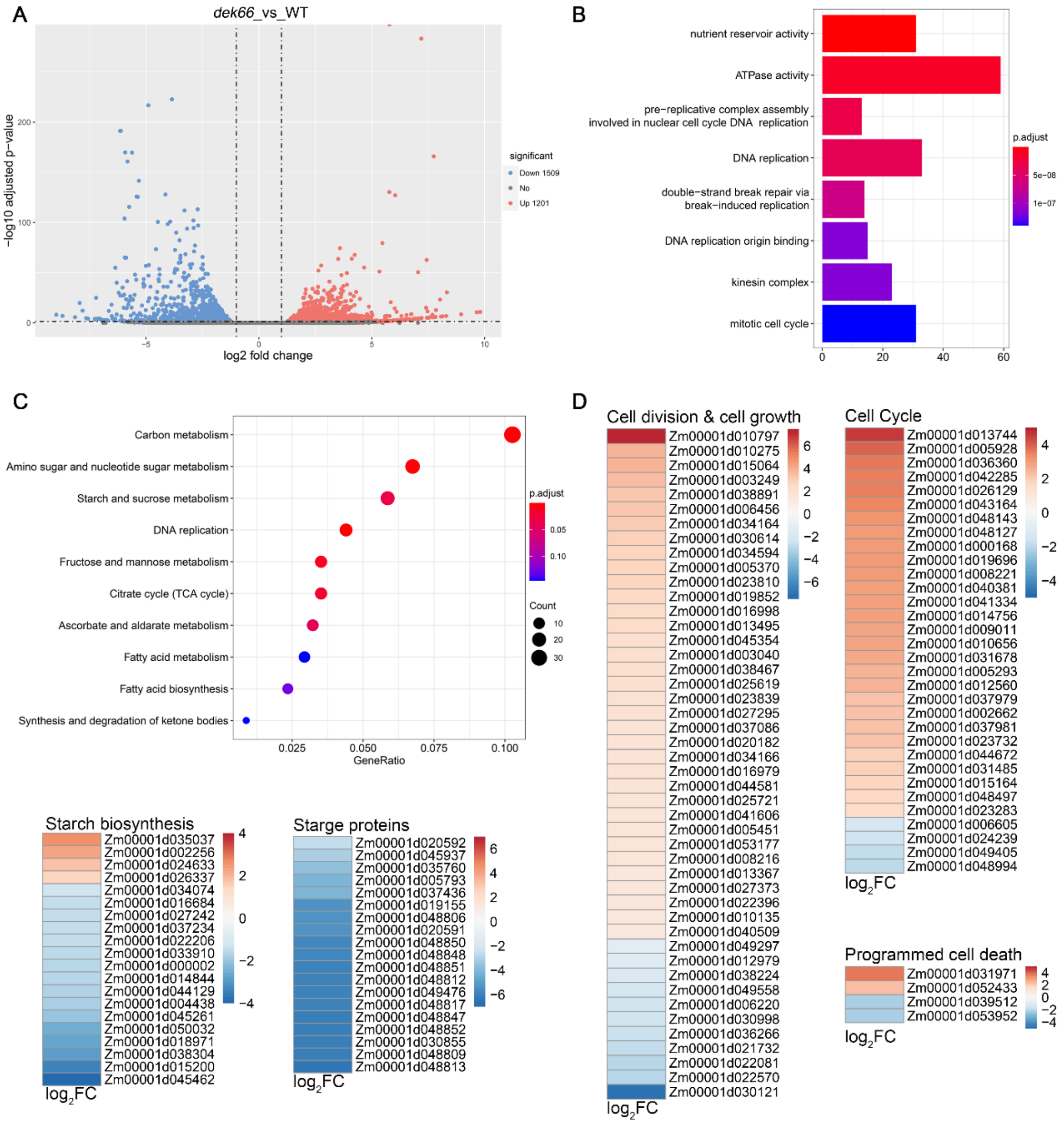

Fig. S9. ***DEK66*** **regulates genes involved in diverse processes.**

(A) Volcano plots were used to visualize RNA-seq data. Each point corresponds to a DEG. Orange and blue plots represent up-regulated and down-regulated genes, respectively. (B) Enriched GO terms in *dek66* and WT endosperm differentially expressed genes. (C) Pathway functional enrichment of DEGs. X axis represents enrichment factor. Y axis represents pathway name. (D) Log_2 fold change heat maps of differentially expressed genes.

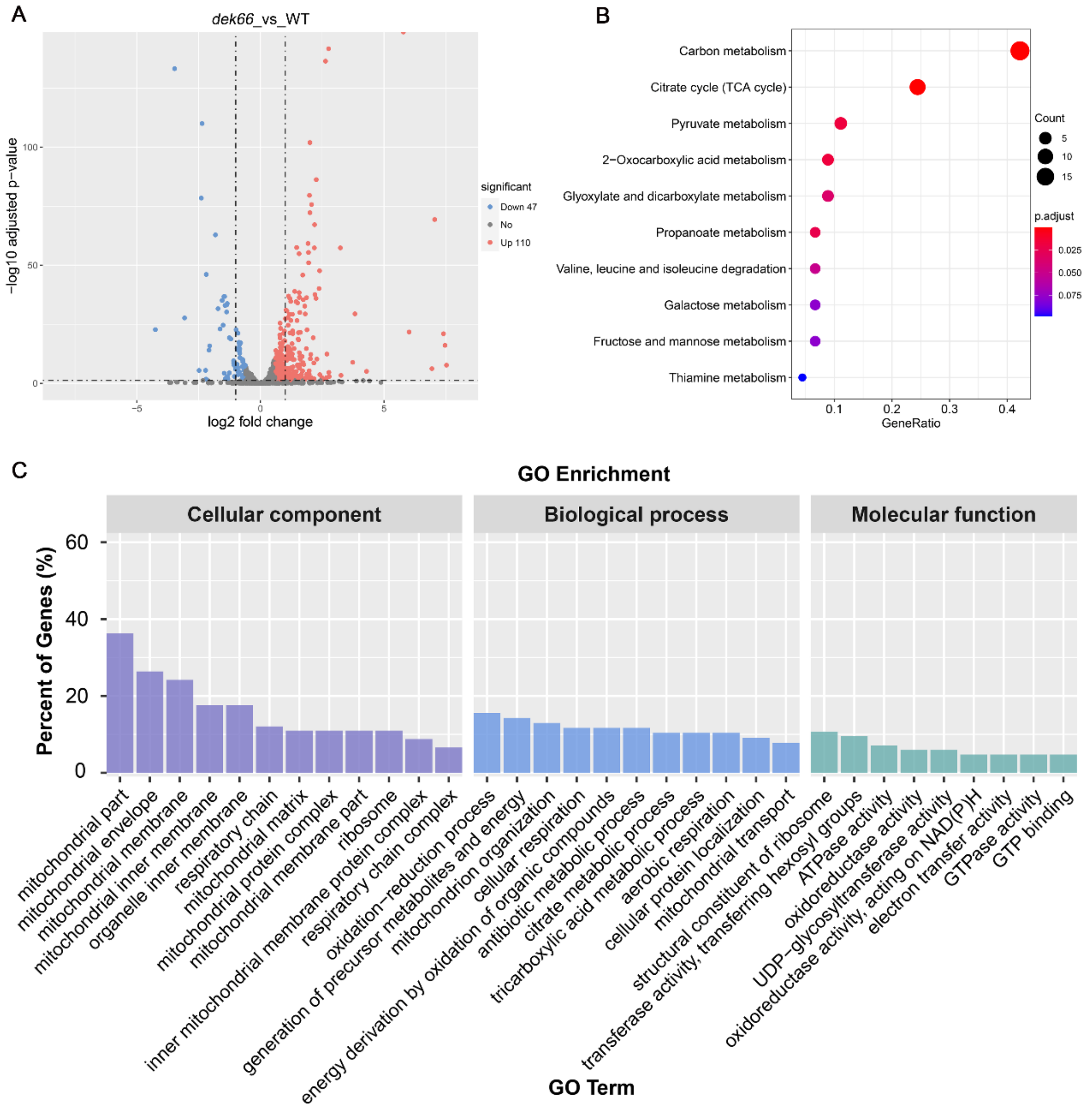

Fig. S10. Analysis of mitochondrial related differentially expressed genes.

(A) Volcano plots were used to visualize RNA-seq data. Each point corresponds to a DEG. Orange and blue plots represent up-regulated and down-regulated genes, respectively. (B) Pathway functional enrichment of DEGs. X axis represents enrichment factor. Y axis represents pathway name. (C) Enriched GO terms in *dek66* and WT endosperm differentially expressed genes.

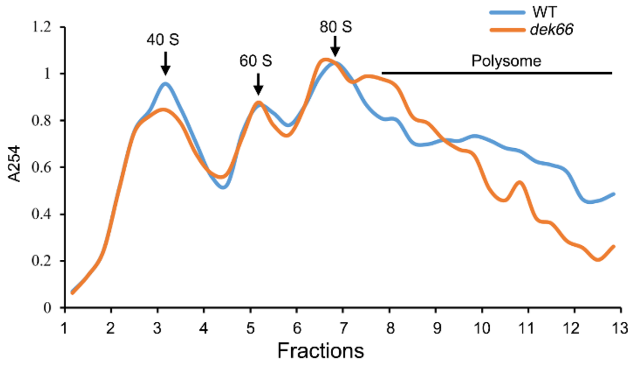

Fig. S11. Polysome profiling assay with sucrose density gradient

The OD_{254} absorption was monitored together with fractionation. The fractions containing 40S, 60S and 80S of ribosome, and polysomes in WT and *dek66* are indicated.

Table S1. List of primers used in this study.

Primer Name	Primer Sequence	Application
umc1191-F	AAGTCATTGCCCAAAGTGTTGC	Mapping-based Cloning
umc1191-R	ACTCATCACCCCTCCAGAGTGTC	
IDP4338-F	CTCGATTCGGAACCAGAGC	
IDP4338-R	GAGGCTCGTACCCTTCACG	
umc1492-F	GAGACCCAACCAAAACTAATAATCTCTT	
umc1492-R	CTGCTGCAGACCATTTGAAATAAC	
umc1771-F	CATCAGGAAGGAAGACGACTAGGA	
umc1771-R	GTGAAATGTTGTTTCCAATGCAAG	
umc1231-F	CTGTAGGGCTGAGAAAAGAGAGGG	
umc1231-R	CGACAACTTAGGAGAACCATGGAG	
chr9-67-F	TCCCATTTCCCCTCTCCCTC	
chr9-67-R	GCGTGGTGTTCATCGAAACC	
chr9-94-F	GTACTGTACGTGGGCAGGAG	
chr9-94-R	CCTTGCCATTTTGCCAACCA	
chr9-178-F	CTCTCCCTCTTGCTCAACCG	
chr9-178-R	TGGATGGGGATCGATGGGTA	
chr9-277-F	TCTTCTCCTGACCTTGGTTCTT	
chr9-277-R	CGGACCTAGCTTTTGTGTTCAA	
13-37-F	CTATTCGCCAACACTCCAGC	
13-37-R	AGTACCTTTGGAGTGTGGGG	
13-48-F	TGTGCTTCCATCCTACTCTGTT	
13-48-R	CCAATGCCTTGTATGGTCGTAG	
13-61-F	GTGTGTGTGTGTGTTTTGGTTC	
13-61-R	TTTCCATTTCCAGTTTCCGACC	
SNP-1-F	TCCCAAATGAGTCAAGCTAGGT	
SNP-1-R	ACGACATACTCTCTGCATGTCT	
DEK66-MT1T2-BsF	AATAATGGTCTCAGGCGCGGCCGTGCGTGGC	CRISPR-cas9-based mutation of *DEK66*
DEK66-MT1T2-F0	GCGGCCGTGCGTGGCCTCTCGTTTTAGAGCTA	
DEK66-MT1T2-R0	TGTGGCGCCCGTACCACGCCGCTTCTTGGTGC	
DEK66-MT1T2-BsR	ATTATTGGTCTCTAAACTGTGGCGCCCGTACC	
DEK66-F	ATCACCACCCTCAGTCGAAG	*dek66* and CRISPR-cas9-based mutation of
DEK66-R	ATTCAGAAGCAGCCGTCAAC	

DEK66-cas9-F	ATCACCACCCTCAGTCGAAG	*DEK66* identification
DEK66-cas9-R	ATTCAGAAGCAGCCGTCAAC	
qRT-*ZmActin*-F	ATGGTCAAGGCCGGTTTCG	qRT-PCR
qRT-*ZmActin*-F	TCAGGATGCCTCTCTTGGCC	
qRT-*DEK66*-F	AGGATATGTACGACCCCGATA	
qRT-*DEK66*-F	CTTGTCCAGACACCTGTACTC	
DEK66-PM999-F	ATGACGACACCGGCGGCGGAGG	35S::DEK66 -eGFP
DEK66-PM999-R	ACAAGGAATCCTTGATGGCTCC	
DEK66-His-F	ATGACGACACCGGCGGCG	Recombinant protein expression
DEK66-His-R	GGCGGTAGGTAATTCTGCG	
US5-1	TTTTAGCCCTGCCTTCATACGC	Identification of *DEK66* overexpression transgenic lines
DEK66-R	ATTCAGAAGCAGCCGTCAAC	
Indel-1F	GACCAATCTCCAGCAACGTC	
Indel-1R	GTCTCCAACAAACGCCATCT	
rps1-F	AAGGTGGGCTTCGGATTATT	qRT-PCR analysis of mitochondrial genes
rps1-R	ACTGAAGAAGATTTGGCGAACG	
rps2A-F	CAGGAAAGATATTTGCCCCA	
rps2A-R	GAGGATAACAAGCGGGAAAAGC	
rps2B-F	TCCCGAGGTTTTGCTCCATG	
rps2B-R	GTTGGTTCTGCTTTGGAGGC	
rps3-F	GGCCTTCAAGCATCCGAAATAC	
rps3-R	CAGAGCGGGACTTCTTTGGTA	
rps4-F	AGAGTTGGGTTCGATTCCCT	
rps4-R	CCTCTGTGCATCTCCCTTATGG	
rps7-F	TTCGTTGGAAAAACCTACGC	
rps7-R	TGATTACATCGCGTTCAGTTCG	
rps7-ct-F	TTGAACCTCTTTCACGCTCA	
rps7-ct-R	ACGTATTGCTTGACGTAAAACCA	
rps12-F	CTAGCTGCTTCCATATCGCC	
rps12-R	CGGATCGGGAGTAACCACTA	
rps12-ct-F	TGTACGGTTCTGTAGAGGGACA	

rps12-ct-R	TAATGCGATATCTCACACCGGG	
rps13-F	TCATGATGATTAAGGGAAGAGTGA	
rps13-R	TTGAATTGAACAGTGTGATTGAT	
rpl16-F	GGTTTTTCCCCACTAACCAA	
rpl16-R	GCAGAAGTTCGAATGGGAAGAG	
nad1-F	GCAACGTCGAAAGGGTCCTG	
nad1-R	CCCAAGCGACCAGACTTAACAT	
nad2-F	GACGGAGGAGAGGAAATGAA	
nad2-R	GCCGGGATCATTAAGAGCATAC	
nad3-F	CTTTCCTATGTCCTTCCCCC	
nad3-R	GAGGAGAGCGAGAGAACGAA	
nad4-F	TTCTCATACCAGACCAACCCAC	
nad4-R	ACGGCCAATTCTCAATTTGTGG	
nad4L-F	CTGACATTCCATGTTTCCGA	
nad4L-R	GAAGAGAACGAAAGGAGAACAGA	
nad5-F	CGCTCGAACATTGTCTGATT	
nad5-R	CCAAGAAGCATCAAACATTTCCG	
nad6-F	ATTCTTTCCGTTTTGTCGAGCC	
nad6-R	ACTGGGAAGATCATAGCGGAGA	
nad7-F	ACTGAATCCCCAATCCTTTGCT	
nad7-R	GAGCATCAACTCCGTTCCTTTG	
nad9-F	ACCCATTTCCATTGTTGTGCTT	
nad9-R	TTACTTCGTCCGCACTTGTTTG	
cob-F	AGGAACCAACGATTCTCTCTTCT	
cob-R	ATACCAGCTAACGAACCGAACC	
cox1-F	GGCTCTTCTCCACTAACCACAA	
cox1-R	TCCCAAATCCACCTATCATCGC	
cox2-F	GTCCTACTTCTGGTGCTGCC	
cox2-R	AGTTTGCTCGTTGAAATGCCAT	
cox3-F	ACGTGATGCTTCTTGGTCAGAT	
cox3-R	AGCAACCGTTTTACTGGCTCTA	

atp1-F	CGTTGCTGGTGAAGAAGCAT	
atp1-R	CAACGGCTTCTGTGATCGAATG	
atp4-F	AGCCACGTGCTCTAATCCTC	
atp4-R	AGTCACAGGTTTTCACTTTTCAGT	
atp6-F	CCAAGTCTCTTTTGGGAGCA	
atp6-R	TCACGGTCTTGAATGCTCCTTC	
atp8-F	GGCAAGGATCCTCAGTCCTA	
atp8-R	CTCACGAGGAATGGAGAGACAG	
atp9-F	CTCACGAGGAATGGAGAGACAG	
atp9-R	TAGTTGCGAAGGAAAAGCGT	
ccmB-F	AGCCGTCGAAGTGAATGAAT	
ccmB-R	ACCTATCATTAAGGGCGTGACG	
ccmC-F	ACCACCAGATCTTCAACAAGGT	
ccmC-R	AGGGGATGTTTTGTTAATGGGAAC	
ccmFN-F	TTCTACGGACCTGCTTCATCTG	
ccmFN-R	GCTCCCGTTCATAGTTCTGCTA	
ccmFC-F	GAGAAGCTCAAATCGAACGG	
ccmFC-R	CTTGCTTTGACCAACACTCCAC	
mat-r-F	TTCAACAGGCAGTCTCACGATC	
mat-r-R	ACTATGTCTCTGTCGCTGACG	
mttB-F	CAGCCCTCTTCACGAACTTGTA	
mttB-R	TATCACAGCAGCTCTTTCCACA	
Zm00001d040805-F	AGGTCCATTTTACCACTACTGG	NADPH oxidase family genes
Zm00001d040805-R	CCATGATGTGAAATACAGCTCG	
Zm00001d007421-F	CACTCATCACCATGATCCAGTC	
Zm00001d007421-R	GAGAATTCCTGCGCAAGTTTG	
Zm00001d007426-F	CCATGATCCAGTCGTTCAACTA	
Zm00001d007426-R	CAGGTTGTACAGCTCGATGTAG	
Zm00001d007430-F	GACTGCAGACTTTCTTCCACAT	
Zm00001d007430-R	TGATCTTCGACAGCTTGTTCTC	
Zm00001d009248-F	TTTTACGGCACAAATGAGTCAG	

Zm00001d009248-R	TTTTGAGCAAACTTACGGAAGG	
Zm00001d009349-F	GTCTTCATAGATGGTCCCTACG	
Zm00001d009349-R	TAACCCAGTAGAAGTAGGCTCT	
Zm00001d023859-F	GAATGTCATCGCCATTCACATG	
Zm00001d023859-R	GATGTGCATGCTCAGGTAGTC	
Zm00001d032079-F	CAGGACTTCAGGAACTACGAC	
Zm00001d032079-R	TTGATGTTGTTGAGGAGGTCTC	
Zm00001d038762-F	ATCGAAGGTTCAAGAGCACATA	
Zm00001d038762-R	TATGTGTCCTACATCACGCTAC	
Zm00001d040974-F	CATCATAAAGGCGGCAATCTAC	
Zm00001d040974-R	CCAATCACCTAACGTACGGATA	
Zm00001d042961-F	CCAAACTGGAGGAAGGTATTCT	
Zm00001d042961-R	ATCCTTCAGTTGTTTTGTGAGC	
Zm00001d043543-F	CTTGGAAGTTCATGCAGTATCG	
Zm00001d043543-R	TTCCCTGCATGGAGGATTATAC	
Zm00001d052653-F	GAACAAGCTTTCGAAGATCACG	
Zm00001d052653-R	CAGGTTGTACAGCTCGATGTAG	
APX1-F	GTCCAAATGTGCATCCTTTCTG	Antioxidant enzymes genes qRT-PCR primers
APX1-R	GAGAAAGGGAAGCTTCTCGTAG	
APX4-F	GAAGAATTACGCCACGCTCAT	
APX4-R	CTAGCAACCTGACGACAATTTC	
CAT1-F	GCCTTGGCATTTCCTTAAGATA	
CAT1-R	GGGAGAAAACAATGTCGGTTC	
GST4-F	CTGGCAGCTGCTATAATAAGGA	
GST4-R	CGCAGTGGATAGGAGTAGAGT	
POX1-F	TCTCTCGCATGCATCCCTCT	
POX1-R	GGCACTGGCACACAACAAAG	
POX3-F	CTCCAAGAACCTCACTATCGAG	
POX3-R	GCTCTTGAGAAGAAATGCGTAG	
SOD3-F	ACTGAGATAGACTAATGCACGG	
SOD3-R	GCATCACCTTGCTCAACATAAA	

SOD4A-F	CAGTGACTTGTGGAGTTGTTTG	
SOD4A-R	CTCTTGGGTGAAAAAGATGGTG	

Table S2. Genetic analysis of the kernels in F_2 population of the segregating ears.

F_2 population	Number of WT	Number of mutants	Actual ratio	Expected ratio	χ^2
dek66/+	2164	718	3.01:1	3:1	0.011
S162/dek66	5998	1956	3.08:1	3:1	0.708
DEK66-KO#1/+	1136	349	3.25:1	3:01	1.778
DEK66-KO#2/+	1435	488	2.94:1	3:01	0.703
